# Pesticide Residues in Vegetables: The Potential Risk Assessment of Endocrine and Reproductive Disruptors for Children and Adults

**DOI:** 10.3390/foods15132336

**Published:** 2026-07-01

**Authors:** Piotr Kaczyński, Piotr Iwaniuk, Izabela Hrynko, Magdalena Jankowska, Ewa Rutkowska, Stanisław Łuniewski, Rafał Konecki, Marcin Pietkun, Weronika Piątek, Elżbieta Wołejko, Damira Absatarova, Bożena Łozowicka

**Affiliations:** 1 Institute of Plant Protection—National Research Institute, Chełmońskiego 22 St., 15-195 Białystok, Poland; p.iwaniuk@iorpib.poznan.pl (P.I.); m.jankowska@iorpib.poznan.pl (M.J.); e.rutkowska@iorpib.poznan.pl (E.R.); r.konecki@iorpib.poznan.pl (R.K.); w.piatek@iorpib.poznan.pl (W.P.); b.lozowicka@iorpib.poznan.pl (B.Ł.); 2 Faculty of Management, Eastern European University of Applied Sciences in Białystok, Ciepła 40 St., 15-472 Białystok, Poland; astwa@astwa.pl; 3 Hydratec, Radziwonika 12 St., 15-166 Białystok, Poland; hydratec006@gmail.com; 4 Department of Chemistry, Biology and Biotechnology, Białystok University of Technology, Wiejska 45A St., 15-351 Białystok, Poland; e.wolejko@pb.edu.pl; 5 Faculty of Natural Science and Technology, Zhetysu University, Zhansugurov 187A St., Taldykorgan 040009, Kazakhstan; mikalok.kz@mail.ru

**Keywords:** vegetables, pesticide residue, endocrine and reproductive disruptors, hazard index

## Abstract

Pesticide residues in vegetables constitute a potential source of exposure to endocrine- and reproductive-disrupting chemicals (EDCs/RDs), particularly in vulnerable populations such as children. This study assessed pesticide occurrence in vegetables from 12 countries worldwide and evaluated associated health risks for children and adults within the framework of European legislation. Of the 390 analyzed samples, 84.7% contained 40 pesticides, including nine non-approved compounds (26% of samples). Fungicides were among the detected groups (81.7%), with boscalid (0.005–0.36 mg kg^−1^) and propamocarb (0.005–0.87 mg kg^−1^) being the most commonly occurring compounds. Multiresidue contamination was observed in 75.7% of samples. Tomatoes and leeks exhibited the highest concentrations, and the European Union maximum residue level (MRL) was exceeded by up to 240% for flonicamid in Chinese cabbage. Propamocarb was the most commonly identified EDC, while tomatoes showed the greatest diversity of these compounds. RDs occurred less frequently, with pyraclostrobin being the most common. Risk assessment resulting from the presence of multiple pesticides in an individual sample expressed as the hazard index (HI) exceeded acceptable levels for BE toddlers consuming tomatoes (1.345 for EDCs and 1.264 for RDs) and leeks (1.010) containing propamocarb. These findings highlight an in-depth toxicological evaluation of the combined effects of multiple hazardous pesticides occurring simultaneously, which may support future legislative measures aimed at improving food safety.

## 1. Introduction

Vegetables play a crucial role in plant-based diets, which have gained popularity. These diets are no longer reserved for vegans and vegetarians but are followed by a considerably larger segment of the population. A turn towards such diets results from the growing awareness of consumers who choose a menu richer in vegetables. The vitamins, trace elements, and nutritional substances contained therein have a favorable effect on the organism and well-being [1]. Furthermore, regular, daily consumption of vegetables in sufficient amounts (approx. 400–500 g) prevents circulatory diseases, cerebral strokes, and certain types of cancer [2].

However, due to the significant relation between the consumption of vegetables and human health, it is vital to pay attention to the quality of the consumed products, particularly the content of toxic and hazardous substances to human life and health [3]. Due to fungal diseases, pests, and weeds, pesticides are often used in vegetable cultivation to ensure a high quality of crops and yields [4]. However, there is a problem with effective plant protection because of the withdrawal of pesticides.

Cyclic withdrawal of hazardous pesticides in the European Union (EU) as a result of more stringent regulatory measures led to problems with effective plant protection. Moreover, the economic profitability of registering pesticides for specific vegetables is insufficient to encourage chemical companies to introduce new products to the market. In this context, chemical protection of vegetables is very problematic and can lead to illegal use of not approved and hazardous pesticides. These compounds negatively affect human health. They are especially dangerous for vegan and vegetarian diets due to the large portions of vegetables consumed and the accumulation of pesticide residues in the body [5]. Exposure of humans to pesticide residues consumed along with food is an inevitable consequence of the use of pesticides in agriculture. It was noted that pesticide residues pose a greater hazardous effect to people following a vegetarian diet, who consume large amounts of vegetables or fruits [6], leading to acute and chronic toxicological consequences. Typical symptoms of acute poisoning are vomiting, seizures, nausea, blindness, rashes, dizziness, diarrhea, death, etc. Chronic effects include cancer, neurological diseases, diabetes, leukemia, and asthma.

Particular attention should be given to hazardous pesticidal endocrine and reproductive disruptors (ECDs/RDs), as these compounds are among the most frequently associated with adverse health effects [7,8]. These endocrine and reproductive disruptors exert their toxicity primarily through mechanisms of toxicity, including oxidative stress, disruption of hormone synthesis and signaling pathways, mitochondrial dysfunction, and alterations in gene expression [9,10,11].

As a consequence of these mechanisms, endocrine-disrupting pesticides alter the function of hormones and affect developmental, reproductive, neurological, and immune disorders [12]. For example, scientific studies confirmed that chlorpyrifos, belonging to the group of endocrine disruptors, causes damage to reproductive organs, the brain, and the thyroid gland, as well as lowering the level of blood thyroxine [13]. Deltamethrin damages sperm and the placenta [14]. Some endocrine disruptors have been shown to cause harmful effects at doses well below the safe acceptable daily intake (ADI) [15].

In the group of reproductive- and developmental-disrupting pesticides, prochloraz causes reproductive malformations in androgen-dependent tissues, decreases circulating estradiol in women, in addition to oocyte atresia, and decreases circulating testosterone in men [16]. It was also reported that pyraclostrobin exposure can result in developmental toxicity and hepatotoxicity [17], while imidacloprid increases the incidence of wavy ribs and a high number of male fetuses [18].

Although the toxic effects of individual pesticides have been widely described in the scientific literature, actual consumer exposure most often results from the simultaneous intake of multiple pesticide residues present in a single food product. Such co-occurrence may lead to additive, synergistic, or antagonistic effects, meaning that the overall toxic effect of the mixture may differ from that of individual compounds assessed separately. This issue is particularly important in the case of pesticides exhibiting similar mechanisms of action, whose combined effects may enhance adverse health outcomes even when the concentrations of individual residues do not exceed the established regulatory limits [19,20].

Due to their widespread occurrence and potential for accumulation even at low concentrations, these compounds pose a significant risk to human health. The majority of compounds belonging to these groups are not approved in the EU, including, e.g., herbicides (prochloraz), fungicides (benthiavalicarb, carbendazim, and dicloran), and insecticides (e.g., chlorpyrifos, imidacloprid, fenbutatin oxide, and diafenthiuron). Occurrences in single raw/processed commodity multi-pesticide residues that pose the same toxicological effect on human health are particularly dangerous due to the risk of synergism in their adverse health effects, leading to cumulative toxicity. Consequently, in-depth and systematic pesticide residue monitoring in vegetables is an essential task to fulfill the full toxicological picture based on cumulative risk assessment, which is important to ensure food safety [21]. Therefore, in accordance with European Union legislation [22], tests are conducted to determine pesticide residue levels, and acute and chronic risks are assessed. According to the European Food Safety Authority’s 2024 report, 96.7% of 86,449 food samples complied with legal pesticide residue limits, continuing the stable trend observed in recent years (96.3% in 2022–2023, 96.1% in 2021, and 94.9% in 2020). The MRL exceedance rate was 3.3%, while the non-compliance rate reached 1.8%, slightly higher than in 2023 (1.5%) but lower than in 2021–2022. Acute and chronic dietary exposure assessments indicated a low overall health risk for most EU population groups and the pesticides evaluated [3]. From a consumer perspective, continuous monitoring of pesticide residues and adherence to food safety regulations remain essential to reduce potential health risks associated with long-term exposure to pesticide mixtures.

The identification and quantification of pesticide residues at ultra-low concentration levels in vegetables is a challenging task due to their complex composition. Therefore, the selection of solvents for extraction and isolation for target analytes, cleanup purifying agents depending on matrix composition, and the mobile phases/gases during instrumental analysis is crucial to achieving a unified, one-step, reliable, and effective multiresidue analytical procedure [23]. Nowadays, a fundamental analytical technique for pesticide residue analysis in such matrices is the combination of the two techniques: gas chromatography and liquid chromatography coupled with tandem mass spectrometry (GC/LC/MS/MS) [5].

The objective of the present innovative and widespread study was to (1) estimate the level of pesticides in the most popular domestic and imported vegetables in hypermarkets in Europe using gas/liquid chromatography tandem mass spectrometry GC/LC/MS/MS; (2) conduct an in-depth toxicological study in diverse populations of children and adults, with particular emphasis on acute health risks for each endocrine- and reproductive-disrupting pesticide separately and for multiple ECDs/RDs in individual vegetable samples evaluated using the hazard index (HI); and (3) identify approved and not approved pesticides and verify their concentration according to applicable European regulations.

## 2. Materials and Methods

### 2.1. Samples

A total of 390 vegetable samples were purchased in hypermarkets in five cities in Poland, each with a population exceeding 200,000, between 10 July and 18 September 2024. The vegetables originated from 8 European countries (Poland, Spain, Italy, France, Germany, The Netherlands, Belgium, and Cyprus) and 4 countries outside Europe (India, Türkiye, Morocco, and the USA). Assortments included 15 commonly consumed species belonging to 6 main groups: Solanaceae (eggplant (10), pepper (10), tomato (70), and potato (80)), Cucurbitaceae (cucumber (40)), Amaryllidaceae (onion (50), garlic (10), and leek (10)), Brassicaceae (broccoli (30), radish (30), and Chinese cabbage (10)), Asteraceae (Romaine lettuce (10) and basil (10)), and Umbelliferae (parsley leaves (10) and celery stalk (10)). Vegetable samples were collected repeatedly according to a predefined sampling plan to ensure a representative number of samples for each commodity. Products were randomly selected from those available for sale and included vegetables originating from different countries. This sampling strategy was designed to provide a realistic representation of consumer exposure to pesticide residues in vegetables available on the market.

For each vegetable sample, 2 kg of product was milled using a laboratory mill (Waring Commercial, Torrington, CT, USA). The homogenized samples were carefully bagged and stored frozen until further analysis.

### 2.2. Pesticide Standards, Reagents, and Materials

Standards of 552 pesticides (237 insecticides, 157 herbicides, 142 fungicides, 11 acaricides, 4 growth regulators, and 1 nematicide) with purities >95.0% were purchased from Dr. Ehrenstorfer Laboratory (Augsburg, Germany) (Table S1). Triphenyl phosphate (TPP), atrazine–d5, carbendazim–d3, and isoproturon–d6 as the internal standards (ISs) were supplied from Sigma–Aldrich (St. Louis, MO, USA).

Individual stock standard solutions (552 pesticides) were prepared at a concentration of around 1000 mg mL^−1^ in acetone. The preparation of working standards solutions (limit of quantification (LOQ), 2LOQ, 10LOQ, 20LOQ, and 100 LOQ mg L^−1^) was obtained by serial dilution of the stock solutions with methanol for LC/MS/MS analysis and with n–hexane: acetone (9:1, *v*/*v*) for GC/MS/MS analysis. The working standard solutions were used for the preparation of matrix-matched standards and the spiking of samples in validation studies.

Internal standards were prepared in the same way as standard solutions. All prepared solutions were kept at about −20 °C in the dark until analysis.

Acetonitrile, methanol, and formic acid were purchased from Merck (Darmstadt, Germany). Acetone and hexane were obtained from J.T. Baker (Edo. De Mexico, Toluca, Mexico). Ultrapure water was derived from a Milli–Q ultrapure water purification system (Millipore, Burlington, MA, USA). QuEChERS kits containing extraction sorbents and salts (4 g MgSO_4_ + 1 g NaCl + 500 mg sodium citrate dibasic sesquihydrate (Na_2_Cit) + 1 g sodium citrate tribasic dihydrate (Na_3_Cit)) and cleanup sorbents (primary–secondary amine—PSA, magnesium sulfate—MgSO_4_, octadecyl—C18, and graphitized carbon black—GCB) were provided by Agilent Technologies (Santa Clara, CA, USA).

### 2.3. Sample Preparation

Vegetable samples were prepared using accredited and validated multiresidue methods based on the EN 15662:2018 standard (QuEChERS) [24]. Sample preparation involved acetonitrile extraction/partitioning followed by dispersive solid-phase extraction (d-SPE) cleanup, while pesticide residues were determined using GC-MS/MS and LC-MS/MS techniques as previously described [21].

**Figure 1:**
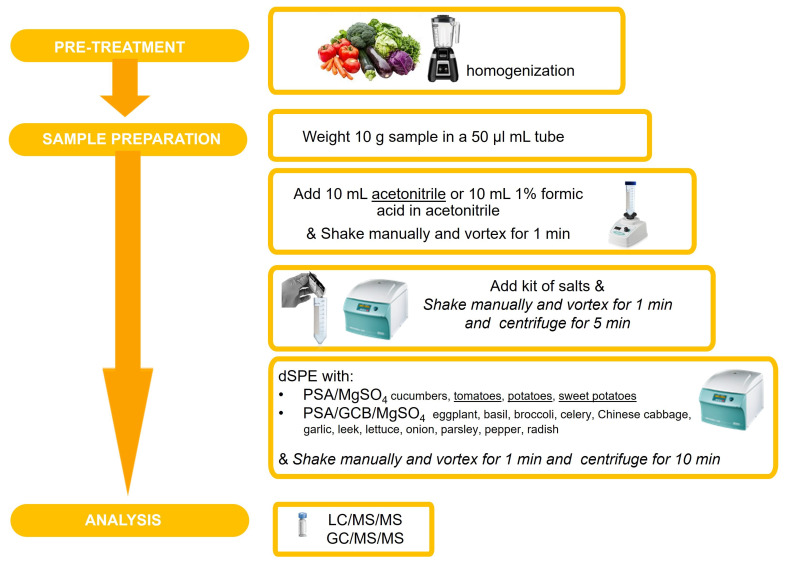
Scheme of procedure to determine 552 pesticides in vegetable samples.

The developed method was externally validated through successful participation in international Proficiency Tests (PTs) organized by the European Union Reference Laboratories (EURLs): EU Proficiency Test on Pesticide Residues in Fruit and Vegetables and EU Proficiency Test for Residues of Pesticides Requiring Single Residue Methods. The PT matrices included dried beans and tomato. Satisfactory z-scores obtained in all tests demonstrated the robustness and fitness-for-purpose of the optimized method for the determination of pesticide residues in vegetables. Detailed PT results are presented in Table S2. Due to their precision and robustness, these methods were used for this study. The scheme of vegetable sample preparation is shown in Figure 1. Detailed validation parameters are provided in the Supplementary Material.

In-house quality control tests were routinely performed to ensure the accuracy and precision of pesticide residue analyses. These procedures included the analysis of procedural blanks, fortified samples, and calibration standards in matrices/solvent at the beginning and end of the sequence, allowing for continuous verification of method performance and the reliability of the obtained results.

### 2.4. GC/LC/MS/MS Analysis

In the study, 552 pesticides were analyzed, of which 255 were determined on an Agilent 7890B GC system coupled to an Agilent 7000B series MS/MS triple quadrupole system (Agilent, Palo Alto, CA, USA). On the other hand, 297 pesticides were determined on an Eksigent Ultra LC–100 system (Eksigent Technologies, Dublin, CA, USA) coupled to a QTRAP 6500 MS/MS triple quadrupole system (AB Sciex instruments, Foster City, CA, USA) (Table S1).

GC and LC system conditions and triple quadrupole system parameters are listed in Table S3, and acquisition parameters in Table S4.

### 2.5. Dietary Acute Risk Assessment

The risk assessment calculations were performed in accordance with EFSA guidance [25]. The dietary acute health risk was conducted utilizing the advanced EFSA Pesticide Residue Intake Model (PRIMo) version 3.1, yielding novel insights for consumers regarding the potential toxicity of pesticides and their exposure during a single meal [26].

Acute risk assessment, according to EFSA methodology, has been considered for the most critical diets of European subpopulations of children and adults, which are different for each raw agricultural commodity. They cover all other European diets that lead to a lower exposure. They are chosen by EFSA according to the greatest consumption data and defined in the calculation model. Other European diets will lead to lower short-term exposure.

Two models of the International Estimated Short-Term Intake (IESTI) calculations were performed under a “worst-case” scenario, incorporating high consumption levels at the 97.5th percentile (large portion, LP). The first model (IESTI) was based on the highest residue (HR) in particular vegetable commodities, while the second one (IESTI_new_) was based on the current maximum residue level (MRL) established by the European Union [22].

These values were then compared to the acute reference dose (ARfD) using the following formula: %ARfD = (IESTI or IESTI_new_/ARfD) * 100%. The IESTI and IESTI_new_ calculation equations, which vary according to the unit weight of each commodity (U), are presented in Table S5. In cases where the acute exposure exceeded 100% of the ARfD, the EFSA PRIMo Model calculated a threshold residue according to guidelines. This value is a residue concentration that would result in 100% of the toxicological reference dose.

Food consumption data used for acute exposure assessments based on the 97.5th percentile consumption (P97.5 consumption) and large portion (LP) were provided by Member States who have derived this information from national food surveys. There is a lack of complete studies for Polish consumers because these studies are available only for the general population. Consumption data for the most critical subpopulations of European adults and children are detailed in Table S6.

For assessing the health risks from combined exposures to multiple pesticides in one sample, the hazard index (HI) was calculated by summing the short-term intakes (IESTI and IESTI_new_) of each endocrine or reproductive pesticidal disruptor and dividing by 100% for two subpopulations: adults and children. If the HI is below one (HI < 1), the adverse effects are no more likely to occur, and exposure is assumed to be safe. The HI is widely used in cumulative risk assessment as it allows for the evaluation of potential health effects arising from simultaneous exposure to multiple chemical substances producing similar toxicological effects. The approach is based on the assumption of dose additivity, whereby the overall effect of a chemical mixture is considered to be the sum of the effects of its individual constituents belonging to the substance groups with the same toxicological mode of action. This method is particularly suitable for assessing cumulative risks associated with substances that affect the same target organs, biological systems, or physiological functions [27,28].

## 3. Results and Discussion

### 3.1. Vegetable Analytical Methods Overview

Vegetables are characterized by various profiles of interfering compounds, including pigments, lipids and fatty acids, sugars and carbohydrates, water, sulphur compounds, alkaloids, phenolic compounds, and organic acids [29]. Optimizing the extraction method for such complex matrices is a crucial step to obtaining a reliable and standardized analytical method.

The literature contains information on the application of different sorbents in the process of purifying extracts from vegetable samples. In the studies by Balkan and Yilmaz [30], the use of PSA, GCB, and MgSO_4_ enabled satisfactory recoveries (80.0–110.0%) for 260 compounds in leafy vegetable samples. Similarly, this sorbent combination proved highly effective in removing interfering substances from fruiting vegetables such as tomato, cucumber, eggplant, and pepper, with recoveries ranging from 68.2% to 108.0% [31]. Comparable recoveries (60.0–129.0%) were obtained by Rutkowska et al. [32], who developed a method for the determination of 590 pesticides. The other commonly proposed cleanup strategies include PSA with MgSO_4_ [33,34] or combinations of PSA, C18, and MgSO_4_ [35]. The results reported by these authors indicate that the appropriate selection of cleanup sorbents plays a crucial role in the efficient removal of matrix interferences in complex plant matrices.

Another huge challenge was to include 552 pesticides characterized by different physicochemical properties (polarity, solubility, volatility, or molecular weight), chemical structure, and thermally unstable compounds that degrade at high temperature (dicofol, captan, or folpet) or at a specific pH (amitraz, dichlofluanid, carbosulfan, and tolylfluanid) in one analytical run [36]. The complex and highly diverse nature of these compounds, together with their large number, significantly complicates multiresidue analysis in terms of both selectivity and sensitivity.

Consequently, proper sample preparation is one of the most critical steps determining the reliability, repeatability, and overall quality of analytical results. Appropriate optimization of extraction and cleanup procedures allows for the effective reduction in matrix interferences, thereby improving the accuracy of determinations. However, due to differences in the content of interfering substances in the tested matrices and the physicochemical properties of the analytes, each case required an individual approach. In this study, a series of experiments was performed to select an appropriate combination of cleanup sorbents, including PSA, GCB, and C18. PSA combined with MgSO_4_ was selected for high-water-content matrices (cucumber, tomato, potato, and sweet potato), whereas a mixture of PSA, GCB, and MgSO_4_ was applied to matrices rich in pigments and alkaloids (eggplant, basil, broccoli, celery, Chinese cabbage, garlic, leek, lettuce, onion, parsley, pepper, and radish).

### 3.2. Validation of Analytical Method

To validate the analytical method, a series of experiments were carried out using vegetable matrices previously checked for the absence of the target pesticides (eggplant, avocado, chickpeas, lettuce, onion, soybean, and tomato), covering linearity, precision, recovery, selectivity, limit of quantification (LOQ), matrix effects (ME), and uncertainty (U). The validation parameters were determined according to the European guidance document [37].

The linearity was evaluated by fitting the chromatographic peak area to the concentration (for six-point LOQ, 2 LOQ, 10 LOQ, 20 LOQ, 50 LOQ, and 100 LOQ mg L^−1^) for each pesticide. All pesticides showed excellent linearity in the tested range in all matrices, with a linear correlation coefficient (R^2^) exceeding 0.99. LOQ is the lowest concentration at which the analyte can be quantitatively detected with a stated accuracy and precision. Precision was assessed from the recovery (R) tests using the following equation, %R = (area response of spiked sample/area response of standard solution) × 100, and it was expressed in terms of relative standard deviation (RSD) at all spiking levels (LOQ, 10LOQ, and 100 LOQ mg L^−1^). The results indicated that the LOQ of all pesticides in vegetables was 0.005/0.01 mg L^−1^, alongside a recovery rate from 60% to 126% in eggplant and soybeans, from 61% to 125% in onions, 61–128% in chickpeas and lettuces, and 61–129% in avocados and tomatoes, while the RSDs were less than 19. The selectivity of the procedure was evaluated by analyzing blank vegetable samples and checking for the absence of interfering peaks in the retention time segment of each target analyte.

The matrix effect (ME) was examined to avoid a false positive result and was evaluated using the following equation: %ME = ((detector response of the standard in matrix/detector response of the standard in the solvent) − 1) × 100. According to values, the ME was divided into three groups: |ME| < 20%—no matrix effect was observed; 20% ≤ |ME| ≤ 50% represented a medium matrix effect; and |ME| > 50% represented a strong matrix effect. Among all of the tested pesticides, about 92% had a weak ME, and the remaining ones had a medium (7%) and strong (1%) ME.

One factor affecting the matrix effect is the physical and chemical properties of the analytes. Compounds that exhibit high polarity, are poorly soluble in water, have a molecular weight of more than 400 g mol^−1^, or contain certain chemical groups are more susceptible to a significant matrix effect. As a result of the study, a strong matrix effect was shown by acephate, a polar compound; propaquizafop and spirodiclofen, compounds with molecular weight above 400 g mol^−1^; and carbosulfan and fluconazole compounds from the group of carbamate and triazole. Given the variable matrix effects for different pesticides, matrix-matched calibrations were used to avoid over—or under–estimation of residue results. Matrix-matched calibration curves were prepared in particular vegetable matrix extracts.

The uncertainties were estimated using a “top–down” empirical model with a confidence coefficient of 95% and taking into account the coverage factor k = 2. Their values rank between 1 and 41%. Each pesticide residue concentration was expressed in mg kg^−1^ with an expanded measurement uncertainty (U) established in the validation step (corresponding to a 95% confidence level and a coverage factor of 2) presented in Table S1.

All validation-obtained parameters were in accordance with the validation and quality control criteria for pesticide residue analysis [37]. All validation parameters are carried out in Table S1.

### 3.3. Pesticides in Vegetables

Ensuring safe food in an era of globalized agricultural trade is a key challenge for public health. The dynamic development of international supply chains means that vegetables available in large-scale retail chains come from diverse geographic regions subject to different legal regulations and pesticide use practices. Most studies referring to the occurrence of pesticides in vegetables concern scientific investigations from Asia or Africa, while research from America and Europe is scarce [5]. Therefore, this is a pioneering investigation revealing results from Europe with the division of domestic and imported vegetables.

A wide range of 552 pesticides was tested in vegetables, including 173 approved in the EU and 379 not approved compounds. Among them, 40 different pesticides were determined in 16 groups of vegetables. A total number of 28 fungicides were determined with 1030 detections (81.7%), varied from 0.005 mg kg^−1^ to 1.1 mg kg^−1^, followed by 12 insecticides with 240 detections (18.3%), in the range of 0.005–0.13 mg kg^−1^. No herbicides were detected in vegetable samples. Results below the LOQ were reported as non-detects (<LOQ). Herbicides are usually applied early in crop production (before sowing, pre-emergence, or at early growth stages) for weed control; therefore, they have more time to degrade, which often results in lower or even non-detectable residues in edible plant tissues [38].

**Figure 2:**
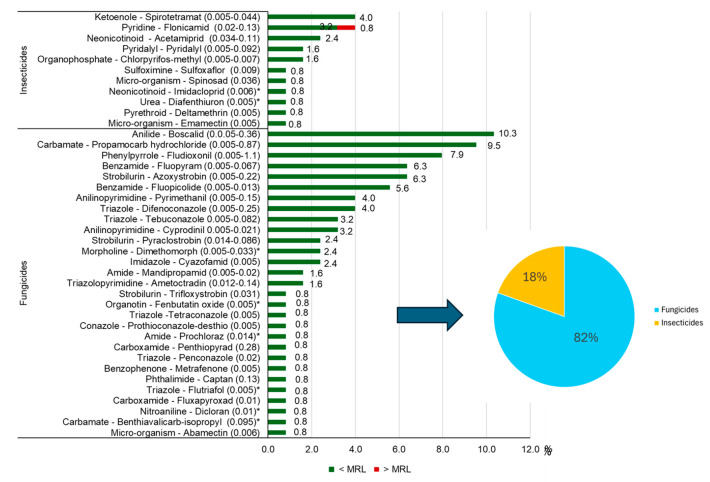
Concentration level (mg kg^−1^) and frequency (%) of insecticide and fungicide detections in vegetable samples (<MRL: below EU maximum residue level; >MRL: above EU maximum residue level). * Pesticides not approved in the EU.

Among chemical groups of detected fungicides, the most commonly determined were anilides (10.3%), carbamates (10.3%), and triazoles (9.6%). Boscalid (10.3%) was detected in the concentration range of 0.005–0.36 mg kg^−1^, followed by propamocarb hydrochloride (9.5%; 0.005–0.87 mg kg^−1^) and fludioxonil (7.9%; 0.005–1.1 mg kg^−1^) (Figure 2). The highest concentration of boscalid in garlic (0.36 mg kg^−1^) and propamocarb (0.87 mg kg^−1^) in parsley leaves were indicated in vegetables from Spain, while fludioxonil (1.1 mg kg^−1^) in sweet potato from the USA (Table S6). Moreover, among chemical groups of insecticides, ketoenole and pyridines were detected the most frequently (4% each). Their representatives were spirotetramat and flonicamid, determined in the concentration range of 0.005–0.044 mg kg^−1^ and 0.02–0.13 mg kg^−1^, respectively (Figure 2). These compounds reached the highest level in broccoli from Spain (spirotetramat) and cucumber from Türkiye (flonicamid) (Table S6).

Metanalysis conducted by Ahmadi et al. [39] indicated that the most frequently detected in vegetables are fungicides, followed by insecticides and herbicides, with a mean concentration of 0.24 mg kg^−1^. As reported by Poornim et al. [40], in vegetables from Sri Lanka, commonly detected pesticides were chlorpyrifos, profenofos, tebuconazole, diazinon, and fipronil. Profenofos and chlorothalonil were identified with the highest concentrations up to 21.98 mg kg^−1^ and 19.37 mg kg^−1^, respectively. In Egyptian vegetables, the most frequently detected pesticides were chlorpyrifos (25.6%), azoxystrobin (17.4%), and acetamiprid (15.4%) [41]. In turn, in vegetables from Bangladesh, chlorpyrifos, dimethoate, diazinon, and malathion were among the most commonly detected pesticides [42]. This study and the abovementioned references confirmed that the most frequently detected pesticides in vegetables from countries other than Europe are not approved in the EU. Therefore, this situation poses a risk of their transfer onto the European market with imported vegetables. The concerning fact is that not approved pesticides were also detected in imported vegetables from European countries.

### 3.4. Multiresidue Samples of Vegetables

**Figure 3:**
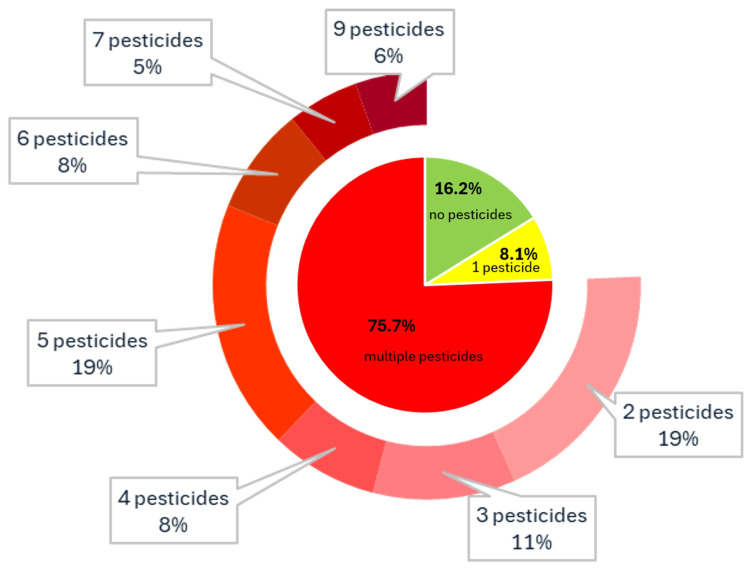
Presence of single and multiple pesticide residues in vegetables.

This study revealed the presence of vegetable samples with multiresidues (75.7%), ranging from two (19%) to nine pesticides (6%) (Figure 3). It was determined that the greatest variety of pesticides was found in a sample of tomato from Poland (9) and leak from Belgium (9), reaching the total concentration 1.038 mg kg^−1^ and 0.207 mg kg^−1^, respectively (Table S7). Pesticide levels ranged from 0.005 mg kg^−1^ to 1.1 mg kg^−1^ for a single pesticide, indicating large variability in analyte concentrations among the samples. Overall, the majority of detected residues were at low concentrations, typically well below established MRL values, suggesting limited acute exposure risk. Moreover, the highest total concentration of pesticides was noticed in parsley leaves (1.35 mg kg^−1^), which had five pesticides (Table S7).

**Figure 4:**
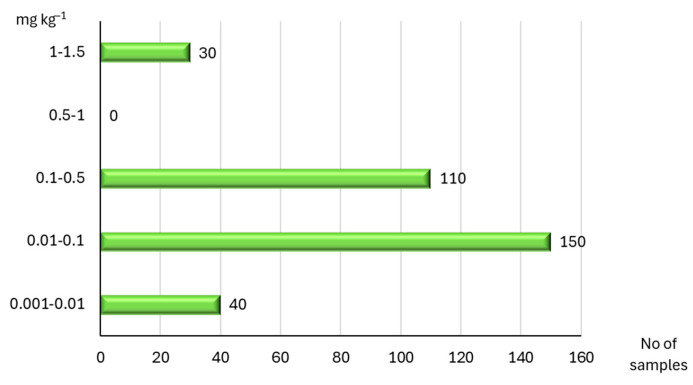
Distribution of the number of samples depending on the range of the sum of pesticide concentration in one sample.

Most pesticide residues were detected at a low level (0.005 mg kg^−1^). According to the range of the sum of detected pesticides in one sample, the highest number of samples (150) were in the concentration range 0.01–0.1 mg kg^−1^, followed by 0.1–0.5 mg kg^−1^ (110) (Figure 4). Based on the metanalysis of Ahmadi et al. [39], the highest total concentration of pesticides belongs to green beans (2.57 mg kg^−1^) and is more than twice as high as in this study (1.038 mg kg^−1^) in the tomato sample from Poland. The examples of GC–LC/MS/MS chromatograms of pesticides in vegetables were indicated in Figures S1 and S2.

Among vegetable samples, 84.7% contained pesticides (Figure 4). The high prevalence of multiresidue samples (75.7%) is of particular toxicological concern, as dietary exposure involves simultaneous ingestion of multiple pesticide residues rather than single compounds. This is particularly relevant in the context of current regulatory frameworks, which are primarily based on individual pesticide evaluations and may not fully capture combined exposure scenarios. Consequently, potential mixture effects remain an important uncertainty in dietary risk assessment. This issue may be especially important for high-consumption groups, such as vegetarians, whose diet is predominantly based on vegetables. Such co-exposure may result in additive or synergistic effects, potentially increasing overall toxicity [19]. The MRL was exceeded for flonicamid (0.03 mg kg^−1^) in the Chinese cabbage sample from Poland by 233% (0.1 mg kg^−1^). Although exceedances were relatively rare (0.8% of samples), their occurrence is still relevant from a regulatory compliance perspective, as even isolated cases indicate potential gaps in good agricultural practice. Table S6 provides the details of detected pesticides. One pesticide residue was detected in 8.1% of samples, including fludioxonil (0.005 mg kg^−1^) in potato from Poland, dimetomorph and mandipropamid in radish from Italy (0.005 mg kg^−1^ and 0.02 mg kg^−1^), and propamocarb hydrochloride in potato from Poland (0.005 mg kg^−1^). The situation described in the report presented by EFSA is somewhat different from what we found in our study: 16.1% of all samples collected in European countries contained one pesticide, while 25.5% of samples were multiresidues [3]. The highest number from vegetable samples with multiple residues was reported in paprika powder (109 samples). According to the 2024 EFSA report from the official control, most of the MRL violations were caused by chlorpyrifos (0.5%). Still, it has been reported in 90 different commodities, the most often being kales and sweet/bell peppers. Poland, Ukraine, India, and Egypt are the countries of origin with the highest findings [3].

Multiple applications of pesticides in the growing season and after harvest (long storage or transport) are effective vegetable protection strategies, but they can lead to the detection of multiple compounds in one sample. Metanalysis conducted in China in 2018–2021 revealed that 29.2% of vegetable samples had pesticide residues, 0.5% exceeded the Chinese level of the MRL, and most samples contained one pesticide [43]. In contrast to this study, Egyptian vegetables had pesticides in 88.4% of samples, with 31.4% above an acceptable MRL, while 66.2% of contaminated samples contained more than one pesticide [41]. Kumar et al. [44] detected pesticide residues in 30% of vegetable samples from the Indian market, with 16% exceeding the MRL. In vegetables from Bangladesh, 29% of samples had pesticide residues below the MRL, but 21% of samples exceeded tolerable MRL values, especially in the case of beans (92%), tomato (78%), eggplant (73%), cucumber (62%), cabbage (50%), and cauliflower (50%) [38]. Among the examined vegetables in Mexico, pesticide residue levels exceeded the EU MRL in spinach, tomato, and chard [45], while in Turkish vegetables, pesticides were detected in 57.6% of samples, and 6.8% exceeded EU MRL values [30]. It indicated that vegetables from Asia, Africa, and Central America exceeded the MRL values in a larger percentage of samples than European vegetables in this study, where exceedances were confirmed in 2.6% of samples.

**Figure 5:**
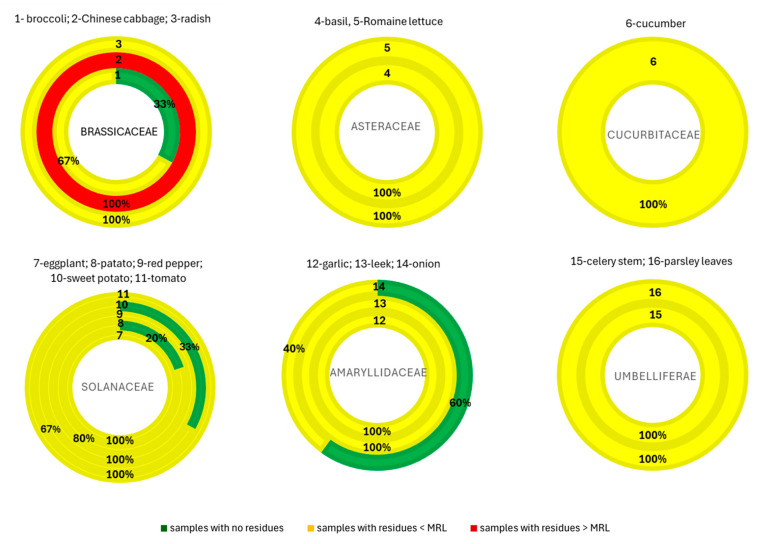
Occurrence of pesticides in six groups of vegetables (Brassicaceae: 1—broccoli; 2—Chinese cabbage; and 3—radish. Asteraceae: 4—basil; 5—Romaine lettuce. Cucurbitaceae: 6—cucumber. Solanaceae: 7—eggplant; 8—potato; 9—red pepper; 10—sweet potato; and 11—tomato. Amaryllidaceae: 12—garlic; 13—leek; and 14—onion. Umbelliferae: 15—celery stem; 16—parsley leaves).

These scientific results indicated that nightshade vegetables contained the highest percentage of samples with residues (38.5%), followed by cabbage vegetables (12.8%), onion vegetables (10.3%), and cucurbit (10.2%). Pesticides were not detected in 30 samples of onion from India, Netherlands, and Spain; 10 samples of broccoli from Spain; 10 samples of sweet potato from Spain; and 10 samples of potato from Poland (Figure 5). In the cabbage vegetables, the most frequently determined pesticides were fluopyram and dimetomorph (29% of samples each), the common pesticides in nightshade vegetables were fludioxonil (41%) and propamocarb hydrochloride (29%), while in onion vegetables it was boscalid (57%) (Table S7).

Among tested vegetables in China in 2018–2021, leafy vegetables had the highest detection rate of pesticide residues (32.9%), the multiple detection rate was 12.2%, and the total pesticide concentration reached 35.700 mg kg^−1^ [43]. Leafy vegetables from Sri Lanka had the highest contamination with pesticides, reaching 43.6% of samples [40]. Among Egyptian vegetables, all samples of tomato, sweet potato, pepper, eggplant, garlic, cucumber, and cabbage had pesticide residues [41]. A similar trend is observed in vegetables from Bangladesh, in which pesticides were frequently determined in cucumber (51%), tomato (41%), cauliflower (31%), eggplant (29%), and beans (23%) [42].

This study and the abovementioned references indicate that vegetables originating from countries outside the EU have a higher percentage of positive samples containing pesticide residues exceeding in some cases 50%, while in selected groups of European vegetables, pesticides were detected in less than 20% of samples (max 18% in tomato samples).

#### Endocrine- and Reproductive-Disrupting Compounds in Multiple Samples

As presented in Table S6, samples of broccoli, Chinese cabbage, celery, lettuce, eggplant, and red pepper did not contain any compounds classified as endocrine-disrupting chemicals (EDCs). The greatest heterogeneity of these compounds has been found in tomato samples, in which propamocarb, penconazole, captan, deltamethrin, and tebuconazole have been detected. A tomato sample originating from Poland has contained as many as four EDCs, propamocarb, captan, deltamethrin, and tebuconazole, in concentrations ranging from 0.005 to 0.41 mg kg^−1^. In contrast, two EDCs—flutriafol and propamocarb—have been identified in a sample of potatoes from Cyprus, at a concentration of 0.005 mg kg^−1^. Chlorpyrifos-methyl has been detected in radishes from Italy (0.008 mg kg^−1^) and in cucumbers from Türkiye (0.005 mg kg^−1^). The cucumber sample additionally contained another EDC—propamocarb (0.016 mg kg^−1^). Propamocarb has been the most frequently detected compound from the EDC group—it has been determined in cucumbers, basil, potatoes, tomatoes, leeks, and onions, in concentrations ranging from 0.005 to 0.87 mg kg^−1^. Penconazole has been detected in a tomato from Italy (0.020 mg kg^−1^).

Similar to EDC compounds, reproductive disruptors (RDs) have also been detected in samples. Pyraclostrobin has been the most frequently detected compound in the RD group—it has been determined in tomato, garlic, and onion, in concentrations ranging from 0.014 to 0.086 mg kg^−1^. Imidacloprid has been detected in a potato from Poland (0.006 mg kg^−1^), dicloran in a sweet potato from the USA (0.10 mg kg^−1^), benthiavalicarb isopropyl in a tomato from Italy (0.095 mg kg^−1^), penthiopyrad in a tomato from Poland (0.28 mg kg^−1^), trifloxystrobin in a tomato from Morocco (0.031 mg kg^−1^), and fenbutation oxide in a tomato from Germany (0.005 mg kg^−1^). Prochloraz, a compound classified as both an EDC and an RD, has been detected in garlic from Spain, alongside other substances that disrupt reproduction (pyraclostrobin) and the endocrine system (tabuconazole) at concentrations of 0.005 and 0.0086 mg kg^−1^. No RDs were found in samples of broccoli, Chinese cabbage, radish, celery, parsley, cucumber, lettuce, basil, eggplant, red pepper, or leek.

In a paper by Jardim and Caldas [46], the presence of EDC pesticides, such as imidacloprid and chlorpyrifos, was demonstrated in samples of various vegetables. In turn, a study by Jankowska et al. [47] showed that fruiting vegetables (including tomatoes and cucumbers) predominantly contained residues of chlorpyrifos, tebuconazole, and deltamethrin, which belong to the group of potential endocrine-disrupting chemicals, as well as imidacloprid and pyraclostrobin, classified as compounds with potential effects on reproductive disorders. Propamocarb was most frequently detected in leafy and cruciferous vegetables, whilst tebuconazole—also classified as an EDC—was most frequently detected in allium vegetables. A review by Khatun et al. [42] showed that vegetables such as tomatoes, cucumbers, and eggplant in Bangladesh frequently contained multiple pesticide residues, including chlorpyrifos and dimethoate, which are associated with potential effects as endocrine disruptors and reproductive disruptors, confirming the high exposure of consumers in non-EU countries to mixtures of these compounds. Likewise, Kumar et al. [44] detected the presence of chlorpyrifos, lambda-cyhalothrin, and pendimethalin in eggplant, peppers, and cauliflowers whilst also noting the frequent co-occurrence of several substances with potential endocrine-disrupting effects in a single sample. In a study by Park et al. [1] on vegetables from the Incheon region in Korea, the presence of pesticide residues was detected in products such as lettuce, cabbage, cucumber, peppers, and radishes. The EDC compounds identified included chlorpyrifos and imidacloprid, with some samples containing several active substances simultaneously.

### 3.5. Not Approved in the EU Pesticides Detected in Vegetables

The study revealed the presence of hazardous, not approved pesticides in the European Union [48,49] in vegetables, which were detected in 26% of all samples tested and concerned five fungicides belonging to carbamates (benthiavalicarb–isopropyl), triazoles (flutriafol), amides (prochloraz), morpholines (dimethomorph), and nitroanilines (dicloran) in the concentration range from 0.005 mg kg^−1^ to 0.095 mg kg^−1^ (Figure 4). Moreover, four not approved insecticides were detected from the chemical group of ureas (diafenthiuron), organotines (fenbutatin oxide), and neonicotinoids (imidacloprid) ranging from 0.005 to 0.006 mg kg^−1^. They were most commonly detected in potatoes (7.7%) (Table S6). Vegetables containing pesticides not approved by the EU were imported from European countries (Germany, Italy, Cyprus, and Spain) and outside the EU (Türkiye and the USA). One sample of tomatoes from Germany and radish from Italy had two not approved compounds. However, one sample of domestic potatoes also included not approved imidacloprid (0.006 mg kg^−1^). The concerning fact is that pesticides not approved in the EU were also detected in European vegetables.

Not approved in the EU pesticides can be present in European vegetables because of food import from third countries, where they are still used for crop protection. Another reason is that European farmers continue to use old stocks of banned products or do not fully comply with restrictions. Moreover, some pesticides, like tebuconazole, are persistent in soil and can be taken up by plants even years after their last application.

### 3.6. Acute Health Risk of the EDCs/RDs Pesticides in Vegetables

Pesticides were classified as endocrine disruptors (EDCs) and/or reproductive disruptors (RDs) based on the information available in the Pesticide Properties DataBase [50], which compiles toxicological and regulatory data.

The risk analysis was focused on 22 compounds of particular toxicological concern, including endocrine disruptors and reproduction/development effects, which have been approved/not approved (NA) in the European Union:

• Nine endocrine disruptors (EDCs):
  (a) Insecticides: chlorpyrifos methyl (NA) and deltamethrin;
  (b) Fungicides: benthiovalicarb (NA), captan, flutriafol (NA), penconazole, propamocarb, prochloraz (NA), and tebuconazole.
• Thirteen reproductive disruptors (RDs):
  (a) Insecticides: fenbutatin oxide (NA), flonicamid, imidacloprid (NA), pyridalyl, and spirotetramat;
  (b) Fungicides: benthiovalicarb (NA), dicloran (NA), dimethomorph, penthiopyrad, pyraclostrobin, prochloraz (NA), prothioconazole-desthio, and trifloxystrobin.

Acute risk assessment was conducted using two complementary approaches: The first was for each endocrine- and reproductive-disrupting pesticide separately, taking into account the calculation of international short-term intake IESTI (input value HR) and IESTI_new_ (input value MRL). The second approach for multiple ECDs/RDs in individual vegetable sample was evaluated using the hazard index (HI) and its calculation based on summing IESTI and IESTInew of each ECD/RD compound.

In the first approach, the short-term exposure was estimated for two populations with regard to 15 vegetable commodities (27 samples) and 22 pesticides. This analysis aimed to evaluate the potential risk associated with the overall occurrence of pesticide residues in the investigated vegetables. Children subpopulations included Belgian toddlers, British infants, and British children 7–10 years; Czech children (4–6 years); and Dutch, German, and Irish children with body weight in the range of 8.7 kg (UK infant)—30.9 kg (UK 7–8 years). Adult subpopulations consisted of British vegetarians, the Dutch general population, German women (14–50 years), and Irish and Lithuanian adults with body weight in the range of 65.8 kg (NL general)—75.2 kg (IE adults). More details are listed in Table S6.

In the case of children, the short-term exposure (IESTI) to a single EDC/RD pesticide, performed with the highest residue level as the input value, was up to IESTI = 18.2% ARfD (pyridalyl/sweet pepper/DE children). When the input value was MRL, new short-term intakes were in the range from IESTI_new_ = 0.1% ARfD (dicloran/sweet potato/IE children) to IESTI_new_ = 151.7% ARfD (pyridalyl/tomato/BE toddlers) (Table 1). For adults, acute exposure was up to IESTI = 10.1% ARfD (flonicamid/Chinese cabbage/UK 15–198 years old). IESTI_new_ varied in the range of 0.1% ARfD (spirotetramat/celery stalk/NL general population and prochloraz/garlic/UK vegetarians) to 96.4% ARfD (pyridalyl/tomato/LT adults) (Table 1).

The threshold residue level (TRL) was determined only in one case, when the short-term intake exceeded 100% (IESTI_new_= 151.7% in BE toddlers). Based on the toxicological data of this study, the TRL was calculated for pyridalyl in tomatoes (TRL = 0.99 mg kg^−1^) (Table 1). For a subpopulation of adults, any pesticide exceeded 100% of the short-term intake. Therefore, the TRL was not calculated.

Intakes for IESTInew were generally higher than IESTI, considering for calculation an MRL level several times higher than the highest pesticide residue concentration detected in the sample as established by the EU (Table 1). Additionally, the acute intakes for children were two–three times higher for the most critical subpopulation of European adults (Table 1), resulting from the differences in the body weight of each group and the varied consumption of vegetables.

Compared to this study, a research investigation of fresh vegetables in Serbia revealed no health risk [51]. In contrast to this study, Khatun et al. [42] determined the health risk for children and adults consuming tomato, eggplant, beans, cauliflower, cabbage, cucumber, and lettuce from Bangladesh. Liang et al. [52] indicated that 11.7% of vegetable samples from China posed a possible health risk in children and adults, calculated for omethoate, phorate, and carbofuran. Shalaby et al. [53] noticed that acute health risk was reliable for chlorpyrifos, cyfluthrin, and dicofol in pepper from Egypt. On the other hand, based on the study from Poland, during a 5-year period (2017–2021), health risk was exceeded only for flonicamid in Brussels sprouts (130.1%) and for chlorpyrifos in rucola (108.1% ARfD) [54]. In another study from Poland during the period 2021–2023, among 849 examined vegetable samples, an acute health risk was calculated only in one sample of dill (120% ARfD for children) [47]. This study and the abovementioned scientific investigation confirm the fact that European vegetables have fewer incidences of acute health risk for children and adults, compared to vegetables originating from countries outside the EU.

### 3.7. Hazard Index (HI)

The hazard index (HI) has been calculated for each sample containing multiple pesticides with similar toxicological effects: endocrine disruptors and reproductive/developmental effects.

In this study, the combined effects of the mixture of pesticides have been calculated by summing intakes IESTI (input value = HR) and IESTI_new_ (input value = MRL) as the effects of each individual ECD/RD compound present in multiple residue samples and expressed as the HI.

Among all detected pesticides in vegetables in this study, 22 compounds have adverse health effects classified as possible or known (Figure S3). The most hazardous is the occurrence of several pesticides with a similar mechanism of action in one sample, which may lead to the accumulation of their toxic effects. According to toxicological studies, detected pesticides are classified as carcinogens, acetylcholinesterase inhibitors, neurotoxicants, respiratory tract irritants, skin irritants, skin sensitizers, eye irritants, and phototoxicants [50]. However, most of them have endocrine and reproductive/developmental-disrupting activity. From the toxicological point of view, the most toxic pesticide detected in this study was chlorpyrifos—not approved in the EU—which was characterized by five adverse health toxicological effects: endocrine disruptor, acetylcholinesterase inhibitor, neurotoxicant, skin irritant, and skin sensitizer. Fortunately, it was determined at a relatively low concentration (0.005–0.008 mg kg^−1^) in two samples of radish and cucumber from Italy and Türkiye (Figure S3).

#### 3.7.1. Endocrine Disruptors (EDCs)

Among all detected pesticides in vegetables, seven fungicides and two insecticides are classified with endocrine-disrupting effects according to the Pesticide DataBase [50]. Referring to the mode of action established by the Fungicide Resistance Action Committee (FRAC) and Insecticide Resistance Action Committee (IRAC), four out of seven fungicidal EDCs detected in this study (flutriafol, penconazole, prochloraz, and tebuconazole) are inhibitors of C14—demethylation in sterol biosynthesis (FRAC group 28). One fungicide, propamocarb, belongs to the carbamates and acts as an inhibitor of phospholipid and fatty acid biosynthesis (FRAC group 28), while the two detected insecticidal EDCs are acetylcholine esterase inhibitors (chlorpyrifos; group 1B IRAC) and sodium channel modulator (deltamethrin; group 3A IRAC).

The calculated HI values (sum of IESTI) range from 0.002 in garlic (IE children, sample No 193) to 0.237 in tomatoes (BE toddlers, sample No 237) in the case of the sub-population of children (Table 2). Higher values of the HI were obtained when summing the IESTI_new_ of an individual ECD (input value MRL) and varied from 0.016 in garlic (IE children, sample No. 193) to 1.345 in tomatoes (sample No. 237).

For adults, the HI was up to 0.064 in tomatoes (LT adults, sample No. 237). Higher values were in the case of the HI, based on the sum of IESTI_new_, and the values ranged from 0.003 in garlic (sample No. 193) to 0.855 in tomatoes (sample No. 237) (Table 2).

#### 3.7.2. Reproductive Disruptors (RDs)

Among all detected pesticides in vegetables, eight fungicides and five insecticides are classified with hazardous reproductive/developmental-disrupting activity according to the Pesticide DataBase (PPDB) [50]. Based on the mode of action, most of these fungicides (penthiopyrad, pyraclostrobin, and trifloxystrobin) are inhibitors of fungal respiration (groups 7 and 11 FRAC), while insecticides are inhibitors of mitochondrial ATP synthase (fenbutatin oxide; group 12B IRAC) and nicotinic acetylcholine receptor agonist (imidacloprid; group 4A IRAC) (Figure S3).

The HI values were in the range of 0.012 in garlic (IE children, sample No. 193) to 0.210 in tomatoes (BE toddlers, sample No. 237) for children (Table 2). Higher HI values were also obtained when the calculation was based on summing IESTInew and varied from 0.051 in garlic to 1.264 in tomatoes.

For adults, the HI ranged from 0.002 in garlic (UK vegetarians) to 0.057 in tomatoes (LT adults). Similarly, in the population of children, higher values of HI were obtained on the basis of the sum of IESTInew and varied from 0.009 in garlic to 0.804 in tomatoes (Table 2).

Currently, the acute health risk is calculated separately for each determined hazardous substance in one sample [55]. Individual components of a mixture may exhibit independent, additive effects when they lead to similar toxicological results, even if their primary mechanisms of action are not identical, or they may interact with each other, resulting in enhanced (synergism) or reduced (antagonism) toxic effects [19]. Therefore, assessing the combined toxic effects of two or more substances in a mixture is a significant challenge in modern toxicology due to the fact that not all the mechanisms of these substances are fully understood.

In this study, particular attention was paid to compounds exhibiting endocrine-disrupting properties (EDCs) and potentially toxic to the reproductive system (RDs) that co-occurred in the analyzed samples. Among the most frequently identified co-occurring substances with others was propamocarb with chlorpyrifos methyl, flutriafol, prothioconazole-destio, and tebuconazole (Table 2).

Propamocarb is a carbamate fungicide with endocrine-disrupting activity. It is a cholinesterase or acetylcholinesterase (AChE) inhibitor; thus, it is highly toxic to the liver and kidneys. It may cause severe allergic skin reactions and secondary sensitization upon repeated contact [56]. Chlorpyrifos is an organophosphate insecticide acting as an acetylcholinesterase (AChE) inhibitor in pests. It is an endocrine disruptor and neurotoxicant. It is highly toxic to the nervous system, digestive system, liver, and respiratory system. It causes headaches, dizziness, confusion, nausea, vomiting, diarrhea, cramps, and muscle weakness [13,57]. Flutriafol induces oxidative stress, apoptosis, and significant alterations in hepatic lipid metabolism, resulting in lipid accumulation and fatty liver lesions. Long-term exposure may therefore pose risks to liver function and metabolic homeostasis [58]. Prothioconazole destio interacts with estrogen and thyroid hormone receptors [59]. Tebuconazole is a triazole fungicide with endocrine-disrupting activity. The compound is a hormone blocker. It disrupts the synthesis of steroid hormones (including progesterone), which may negatively impact reproduction. It affects developmental toxicity, reproductive toxicity, mutagenicity, carcinogenicity, and neurotoxicity [60].

**Figure 6:**
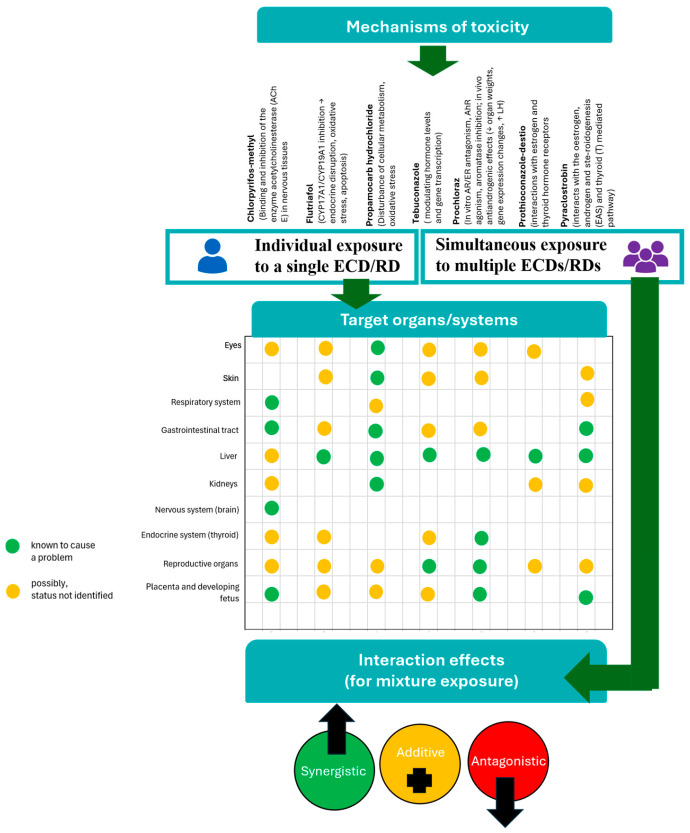
Proposed mechanisms of toxicity of endocrine- and reproductive-disrupting pesticides and their target organs/systems [13,56,57,58,59,60].

Although these compounds exhibit distinct modes of action, toxicological evidence suggests that they may affect common target organs and systems in the human body, particularly the liver, kidney, and brain, affecting the endocrine and reproductive system. Propamocarb has been associated with hepatotoxicity, whereas tebuconazole and flutriafol may interfere with steroid hormone homeostasis and exert hepatotoxic effects (Figure 6). Therefore, their simultaneous occurrence may represent a potential source of cumulative exposure and supports the application of a cumulative risk assessment approach.

Our model of cumulative risk assessments is based on one of EFSA’s recommendations [61]. The EFSA concept has been commonly applied to evaluate chemicals that have the same toxic endpoint through the same mechanism of action, causing the same toxic effects in tissues, organs, and physiological systems, which can produce joint, cumulative toxicity. EFSA classifies pesticides into cumulative assessment groups based on identification of a common target organ and the phenomenological effect [62]. Substances in the first criteria are grouped based on their effects on a general target organ or system, for example, the nervous system, thyroid, acetylcholinesterase, etc. Thus, in our work, we decided to perform a cumulative risk assessment for a group of substances indicating endocrine-disrupting and reproductive/developmental effects. In this study, the combined effects of the mixture of pesticides have been simply summed as the effects of each individual compound according to Kar and Leszczynski [63].

These findings indicate the need for legislative changes that would incorporate the assessment of cumulative exposure and potential acute health risks arising from the simultaneous presence of multiple hazardous substances in a single sample.

## 4. Conclusions

This study highlights a significant issue in the global food supply chain related to the presence of pesticide residues in vegetables. Despite increasing globalization of the vegetable market, pesticide regulations remain inconsistent across countries, particularly outside the European Union, which contributes to food safety concerns. The detection of non-approved pesticides in the EU and exceedances of maximum residue levels (MRLs) indicate ongoing regulatory and monitoring challenges. Acute health risk assessments reveal that children are the most susceptible consumer group, highlighting the urgent need for enhanced surveillance and further investigation into the long-term health effects of these contaminants. Several detected compounds exhibit hazardous properties, including endocrine-disrupting and reproductive toxicity, and contribute most significantly to the overall hazard index. However, considering the broad scope of the dataset, the results should be interpreted as indicative, requiring further validation through continued monitoring. From a risk mitigation perspective, stricter enforcement of pesticide regulations, improved international harmonization of standards, and enhanced monitoring of imported vegetables are recommended. In addition, future research should focus on the combined toxicological effects of multiple pesticide residues, as synergistic interactions may increase overall health risks and are not yet sufficiently understood.

## Figures and Tables

**Table 1:** Short-term acute health risk for the most critical EU subpopulations of children and adults consuming vegetables.

Sample No. and Country of Origin	Commodities	The Highest Residue (Input for IESTI) mg kg^−^^1^	MRL (Input for IESTI_new_) mg kg^−1^	Pesticide	ARfD	Not Approved in UE	EDCs	RDs	Children		Adults		Children Subpopulation	Adult Subpopulation
									IESTI in % ARfD	IESTI_new_ in % ARfD	IESTI in % ARfD	IESTI_new_ in % ARfD		
105 Spain	Broccoli	0.044	1	spirotetramat	1	YES		?	0.2	2.5	0.1	1.9	BE toddlers	NL general population
220 Italy	Radish	0.008	0.01	chlorpyrifos methyl	0.005	YES	+		3.9	2.1	1.7	0.9	NL children	DE women 14–50 years old
		0.033	1.5	dimethomorph	0.6	YES		?	0.1	2.6	0.1	1.1		
294 Italy		0.005	1.5	dimethomorph	0.6	YES		?	0.0	2.6	0.0	1.1		
46 Spain	Tomato	0.002	4	propamocarb	1	NO	+		0.0	12.1	0.0	7.7	BE toddlers	LT adults
62 Italy		0.095	0.3	bentiovalicarb	0.1	YES	+	+	5.5	9.1	1.5	5.8		
		0.020	0.1	penconazole	0.5	NO	+	?	0.2	0.6	0.1	0.4		
22 Poland		0.014	0.3	pyraclostrobin	0.03	NO		+	2.7	30.3	0.7	19.3		
237 Poland		0.405	4	propamocarb	1	NO	+		2.4	12.1	0.6	7.7		
		0.005	0.07	deltamethrin	0.01	NO	+	?	2.9	21.2	0.8	13.5		
		0.131	1	captan	0.3	NO	+		2.5	10.1	0.7	6.4		
		0.284	2	penthiopyrad	0.75	NO		+	2.2	8.1	0.6	5.1		
		0.082	0.9	tebuconazole	0.03	NO	+	?	15.9	91.0	4.3	57.9		
		0.004	2	spirotetramat	1	YES		?	0.0	6.1	0.0	3.9		
253 Netherlands		0.005	1.5	pyridalyl	0.03	YES		?	1.0	**151.7**	0.3	96.4		
261 Marocco		0.021	0.9	tebuconazole	0.03	NO	+	?	4.1	91.0	1.1	57.9		
		0.031	0.7	trifloxystrobin	0.5	NO		+	0.4	4.2	0.1	2.7		
302 Germany		0.005	0.01	fenbutation oxide	0.1	YES		+	0.3	0.3	0.1	0.2		
54 Türkiye	Cucumber	0.016	5	propamocarb	1	NO	+		0.1	19.7	0.0	8.3	CZ 4–6 years old	NL general population
		0.003	0.01	chlorpyrifos methyl	0.005	YES	+		3.9	7.9	1.7	3.3		
243 Poland		0.003	5	propamocarb	1	NO	+		0.0	19.7	0.0	8.3		
277 Poland		0.043	5	propamocarb	1	NO	+		0.3	19.7	0.1	8.3		
85 Türkiye	Sweet pepper	0.092	0.9	pyridalyl	0.03	YES		?	18.2	76.5	5.0	21.0	DE children	UK vegetarians
108 Spain	Celery stalk	0.002	4	spirotetramat	1	YES		?	0.0	0.2	0.0	0.1	NL toddler	NL general population
129 Cyprus	Potato	0.005	0.3	propamocarb	1	NO	+		0.1	2.0	0.0	0.9	UK infants	UK vegetarians
		0.005	0.01	flutriafol	0.05	YES	+	?	1.5	1.3	0.3	0.6		
368 Cyprus		0.001	0.3	propamocarb	1	NO	+		0.0	2.0	0.0	0.9		
382 Poland		0.007	0.3	propamocarb	1	NO	+		0.1	2.0	0.0	0.9		
		0.006	0.01	imidacloprid	0.08	YES		+	1.2	0.8	0.2	0.4		
394 USA	Sweet potato	0.01	0.01	dicloran	0.025	YES		+	0.2	0.1	0.8	0.4	IE children	IE adults
141 Spain	Lettuce	0.036	7	spirotetramat	1	YES		?	0.1	16.0	0.0	5.1	NL children	NL general population
152 Belgium	Leek	0.007	20	propamocarb	1	NO	+		0.0	50.5	0.0	11.6	BE toddlers	IE adults
		0.005	0.06	prothioconazole–destio	0.01	NO		+	2.9	15.2	0.7	3.5		
		0.002	0.6	tebuconazole	0.03	NO	+	?	0.4	50.5	0.1	11.6		
		0.029	1.5	dimethomorph	0.6	YES		?	0.3	6.3	0.1	1.5		
170 Poland	Onion	0.006	0.4	spirotetramat	1	YES		?	0.0	0.4	0.0	0.3	BE toddlers	UK vegetarians
286 France		0.009	2	propamocarb	1	NO	+		0.0	1.9	0.0	1.3		
		0.014	1.5	pyraclostrobin	0.03	NO		+	1.1	48.7	0.7	31.8		
193 Spain	Garlic	0.086	0.3	pyraclostrobin	0.03	NO		+	1.0	3.5	0.2	0.6	IE children	UK vegetarians
		0.014	0.03	prochloraz	0.025	YES	+	+	0.2	0.4	0.0	0.1		
		0.004	0.1	tebuconazole	0.03	NO	+	?	0.0	1.2	0.0	0.2		
204 Poland	Basil	0.003	30	propamocarb	1	NO	+		0.0	2.2	0.0	0.4	DE children	NL general population
211 Spain	Parsley	0.872	30	propamocarb	1	NO	+		0.1	3.3	0.1	3.6	UK 7−10 years old	UK vegetarians
112 Poland	Chinese cabbage	0.1	0.03	flonicamid	0.025	YES		+	12.9	2.3	10.1	1.8	BE toddlers	UK 15-18 years old

EDCs—endocrine disruptors; RDs—reproductive disruptors; HR—highest residue (input value for IESTI); ARfD—acute reference dose (mg kg^−1^ bw day^−1^); MRL—maximum residue level (mg kg^−1^) (input value for IESTI new); LP—large portion; bw—body weight (kg); IESTI—International Estimated Short-Term Intake (mg kg^−1^ bw day^−1^); IESTI in % ARfD; TRL—threshold residue level (mg kg^−1^). Bold font indicates not acceptable values of IESTI. BE—Belgium, CZ—Czech, DE—German, IE—Irish, LT—Lithuanian, NL—Dutch, and UK—British. +—known; ?—possible.

**Table 2:** Acute health risk assessment of endocrine-disrupting and reproductive toxic pesticides in vegetable samples for children and adults expressed as hazard index.

Sample No.	Commodity or Group of Commodities to Which the MRL Applies	Input Value for IESTI (HR)	Input Value for IESTI _new_ (MRL)	Pesticide	EDCs	RDs	Children					Adults			
							ECDs		RDs			ECDs		RDs	
							HI					HI			
							(IESTI)	(IESTI_new_)		(IESTI)	(IESTI_new_)	(IESTI)	(IESTI_new_)	(IESTI)	(IESTI_new_)
54	Cucumber	0.016	5	propamocarb	+		0.040 (4.0%)	0.276 (27.6%)				0.017 (1.7%)	0.116 (11.6%)		
		0.003	0.01	chlorpyrifosmethyl	+										
62	Tomato	0.095	0.3	bentiovalicarb	+	+	0.057 (5.7%)	0.097 (9.7%)		0.057 (5.7%)	0.097 (9.7%)	0.016 (1.6%)	0.062 (6.2%)	0.016 (1.6%)	0.062 (6.2%)
		0.02	0.1	penconazole	+	?									
237		0.405	4	propamocarb	+		0.237 (23.7%)	**1.345** **(134.5%)**		0.210 (21.0%)	**1.264** **(126.4%)**	0.064 (6.4%)	0.855 (85.5%)	0.057 (5.7%)	0.804 (80.4%)
		0.005	0.07	deltamethrin	+	?									
		0.131	1	captan	+										
		0.284	2	penthiopyrad		+									
		0.082	0.9	tebuconazole	+	?									
		0.004	2	spirotetramat		?									
261		0.021	0.9	tebuconazole	+	?				0.044 (4.4%)	0.953 (95.3%)			0.012 (1.2%)	0.606 (60.6%)
		0.031	0.7	trifloxystrobin		+									
129	Potato	0.005	0.3	propamocarb	+		0.016 (1.6%)	0.033 (3.3%)				0.003 (0.3%)	0.016 (1.6%)		
		0.005	0.01	flutriafol	+	?									
152	Leek	0.007	20	propamocarb	+		0.004 (0.4%)	**1.010** **(101.0%)**		0.036 (3.6%)	0.720 (72.0%)	0.001 (0.1%)	0.232 (23.2%)	0.009 (0.9%)	0.166 (16.6%)
		0.005	0.06	prothioconazole–destio		+									
		0.002	0.6	tebuconazole	+	?									
		0.029	1.5	dimethomorph		?									
193	Garlic	0.086	0.3	pyraclostrobin		+	0.002 (0.2%)	0.016 (1.6%)		0.012 (1.2%)	0.051 (5.1%)	0(0.0%)	0.003 (0.3%)	0.002 (0.2%)	0.009 (0.9%)
		0.014	0.03	prochloraz	+	+									
		0.004	0.1	tebuconazole	+	?									

HI—hazard index; EDCs—endocrine disruptors; RDs—reproductive disruptors; HR—the highest residues (mg kg^−1^) (input value for IESTI); MRL—maximum residue level (mg kg^−1^) (input value for IESTI_new_); IESTI—International Estimated Short-Term Intake (mg kg^−1^ bw day^−1^); IESTI in % ARfD; bold font indicates not acceptable values of IESTI; +—known; ?—possible.

## Data Availability

The original contributions presented in this study are included in the article/supplementary material. Further inquiries can be directed to the corresponding authors.

## Supplementary Materials

The following supporting information can be downloaded at https://www.mdpi.com/article/10.3390/foods15132336/s1. Figure S1. Example of GC−MS/MS chromatogram: (a) standard of azoxystrobin in parsley leaves matrix at 0.005 mg kg^−1^, (b) parsley leaves sample fortified with azoxystrobin at the level of 0.005 mg kg^−1^, and (c) in parsley leaves sample with azoxystrobin residue (0.22 mg kg^−1^). Figure S2. Example of LC−MS/MS chromatogram in cucumber samples: acetamiprid 0.034 mg kg^−1^, cyazofamid 0.005 mg kg^−1^, and propamocarb 0.016 mg kg^−1^. Figure S3. Detailed toxicological effects of detected pesticides in vegetable samples. Table S1. Determined pesticides and validation parameters: recoveries (%), relative standard deviation (RSD%), correlation coefficient (R2), limits of quantification (LOQs), matrix effect (ME%), and expanded uncertainties (U%) of 552 pesticides in eggplant, avocado, chickpea, lettuce, onion, soyabean, and tomato (Excel file). Table S2. Results of participation in proficiency testing. Table S3. Chromatographic conditions and the triple quadrupole system parameters of the LC− and GC−MS/MS instrument. Table S4. Acquisition parameters analyzed pesticides and internal standards (ISs). Table S5. Short-term risk assessment equations for particular vegetable commodities. Table S6. Consumption data of particular vegetable commodities for the most critical subpopulation of children and adults. Table S7. Detailed summary of detected pesticides in selected vegetable samples.

## Abbreviations

The following abbreviations are used in this manuscript:

MRL	Maximum residue level
HI	Hazard index
ECDs/RDs	Endocrine/reproductive disruptors
GC/LC/MS/MS	Gas chromatography and liquid chromatography coupled with tandem mass spectrometry
TPP	Triphenyl phosphate
IS	Internal standards
PSA	Primary Secondary Amine
C18	Octadecyl
MgSO_4_	Magnesium sulfate
GCB	Graphitized carbon black
PRIMo	Pesticide Residue Intake Model
IESTI	International Estimated Short-Term Intake
LP	Large Portion
ARfD	Acute reference dose
LOQ	Limit of quantification
ME	Matrix effects
U	Uncertainty
R	Recovery
NA	Not approved
HR	Highest residue
TRL	Threshold residue level
bw	Body weight
FRAC	Fungicide Resistance Action Committee
IRAC)	Insecticide Resistance Action Committee
PPDB	Pesticide DataBase
RDS	Relative standard deviation
d-SPE	Dispersive solid-phase extraction
