# Supplementary material for: Pesticide Residues in Vegetables: The Potential Risk Assessment of Endocrine and Reproductive Disruptors for Children and Adults
Source: Foods. 2026 Jul 1;15(13):2336. doi: 10.3390/foods15132336 (PMC13361924; doi:10.3390/foods15132336)
Supplement: Supplementary file 1 [file foods-15-02336-s001.zip › Supplementary material.pdf]

Supplementary Material

Figure S1. Example of GC−MS/MS chromatogram a) standard of azoxystrobin in parsley leaves matrix at 0.005 mg kg^{-1}, b) parsley leaves sample fortified with azoxystrobin at the level of 0.005 mg kg^{-1}, c) in parsley leaves sample with azoxystrobin residue (0.22 mg kg^{-1}).

Figure S2. Example of LC−MS/MS chromatogram in cucumber samples: acetamiprid 0.034 mg kg^{-1}, cyazofamid 0.005 mg kg^{-1}, propamocarb 0.016 mg kg^{-1}.

Figure S3. Detailed toxicological effects of detected pesticides in vegetable samples.

Table S1. Determined pesticides and validation parameters: recoveries (%), relative standard deviation (RSD%), correlation coefficient (R2), limits of quantification (LOQ), matrix effect (ME%) and expanded uncertainties (U%) of 552 pesticides in eggplant, avocado, chickpea, lettuce, onion, soyabean, and tomato (excel file).

Table S2. Results of participation in proficiency testing.

Table S3. Chromatographic conditions and the triple quadrupole system parameters of LC− and GC−MS/MS instrument.

Table S4. Acquisition parameters analyzed pesticides and internal standards (ISs).

Table S5. Short−term risk assessment equations for particular vegetable commodities.

Table S6. Consumption data of particular vegetable commodities for the most critical sub−population of children and adults.

Table S7. Detailed summary of detected pesticides in selected vegetable samples.

MRM (344.1>329) MRM (344.1>182.9) MRM (344.1>171.9)

a)

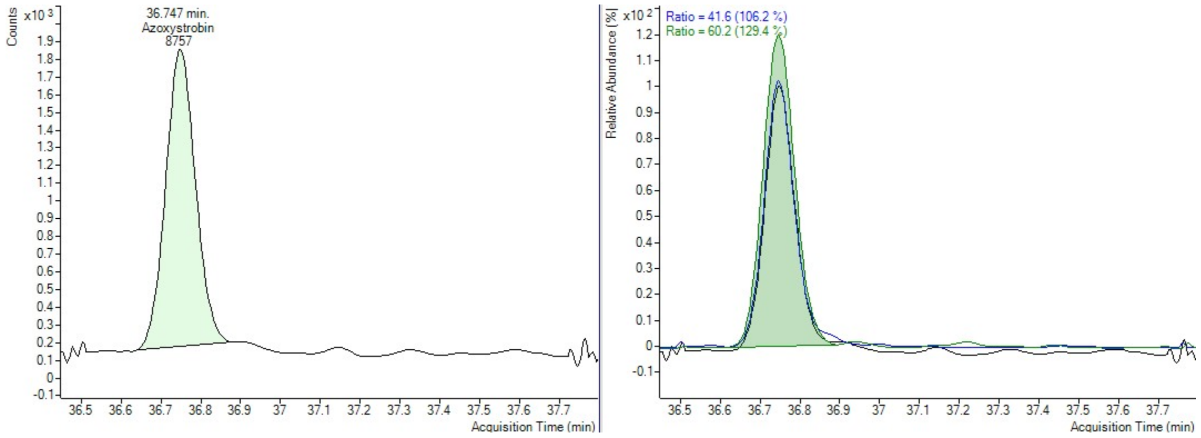

MRM (344.1>329) MRM (344.1>182.9) MRM (344.1>171.9)

b)

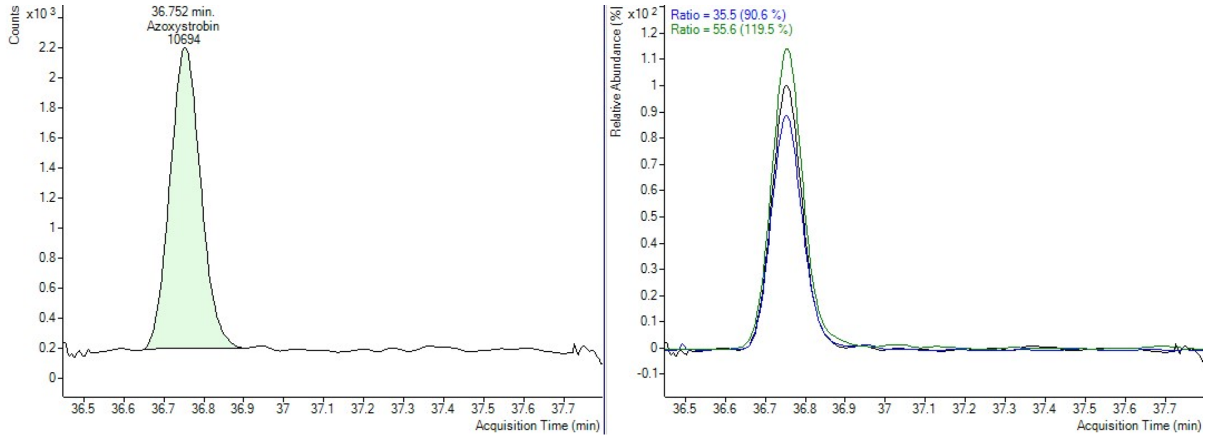

c)

MRM (344.1>329) MRM (344.1>182.9) MRM (344.1>171.9)

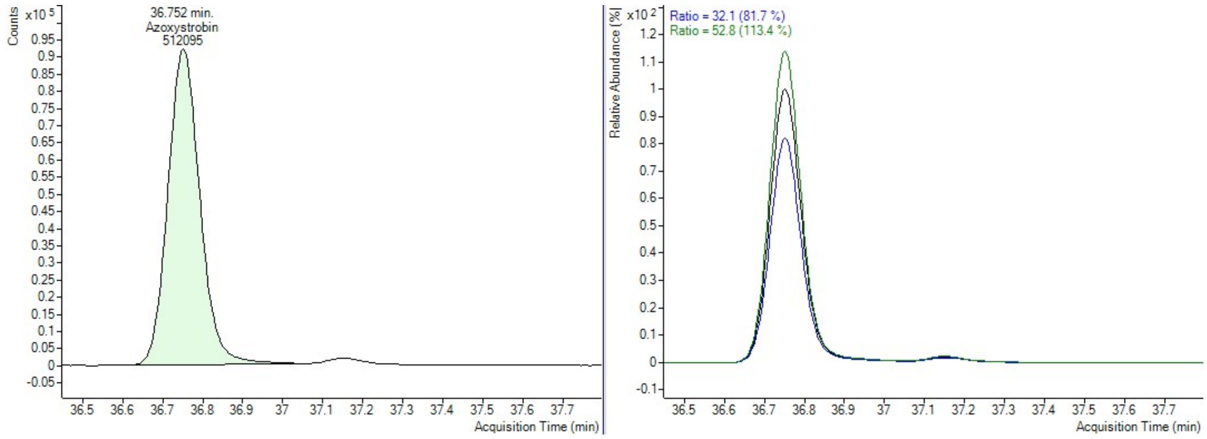

Figure S1. Example of GC−MS/MS chromatogram a) standard of azoxystrobin in parsley leaves matrix at 0.005 mg kg^{-1}, b) parsley leaves sample fortified with azoxystrobin at

the level of 0.005 mg kg^{-1}, c) in parsley leaves sample with azoxystrobin residue (0.22 mg kg^{-1}).

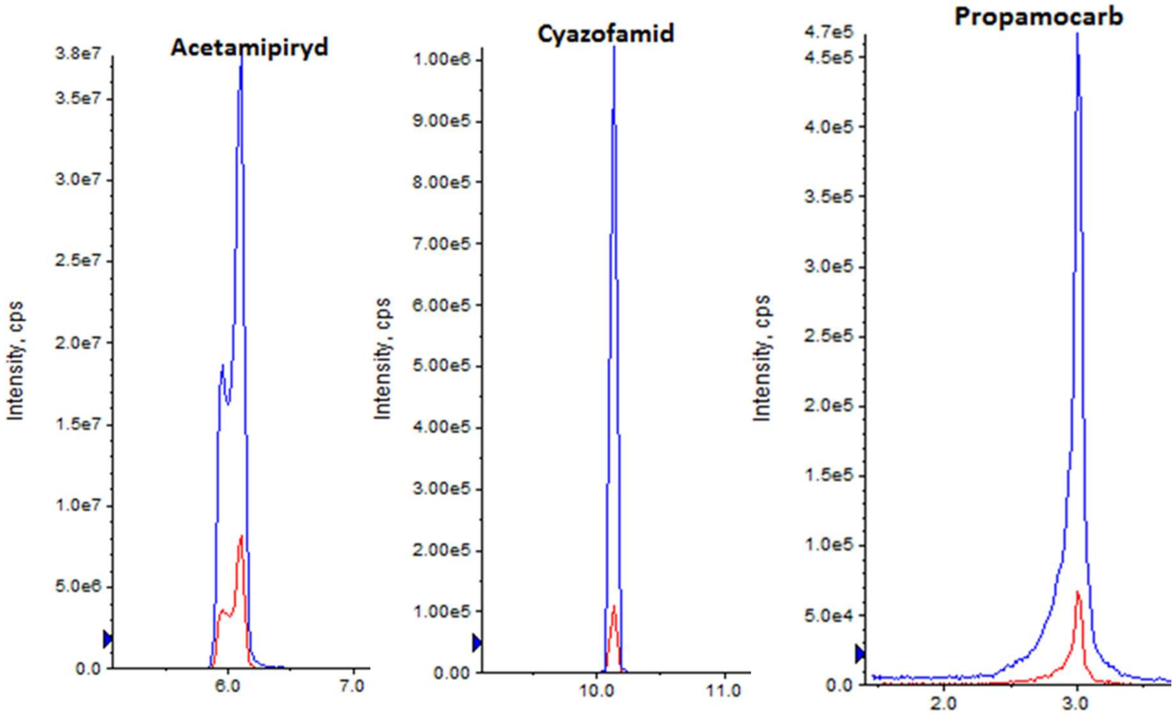

Figure S2. Example of LC−MS/MS chromatogram in cucumber samples: acetamiprid 0.034 mg kg^{-1}; cyazofamid 0.005 mg kg^{-1}, propamocarb 0.016 mg kg^{-1}.

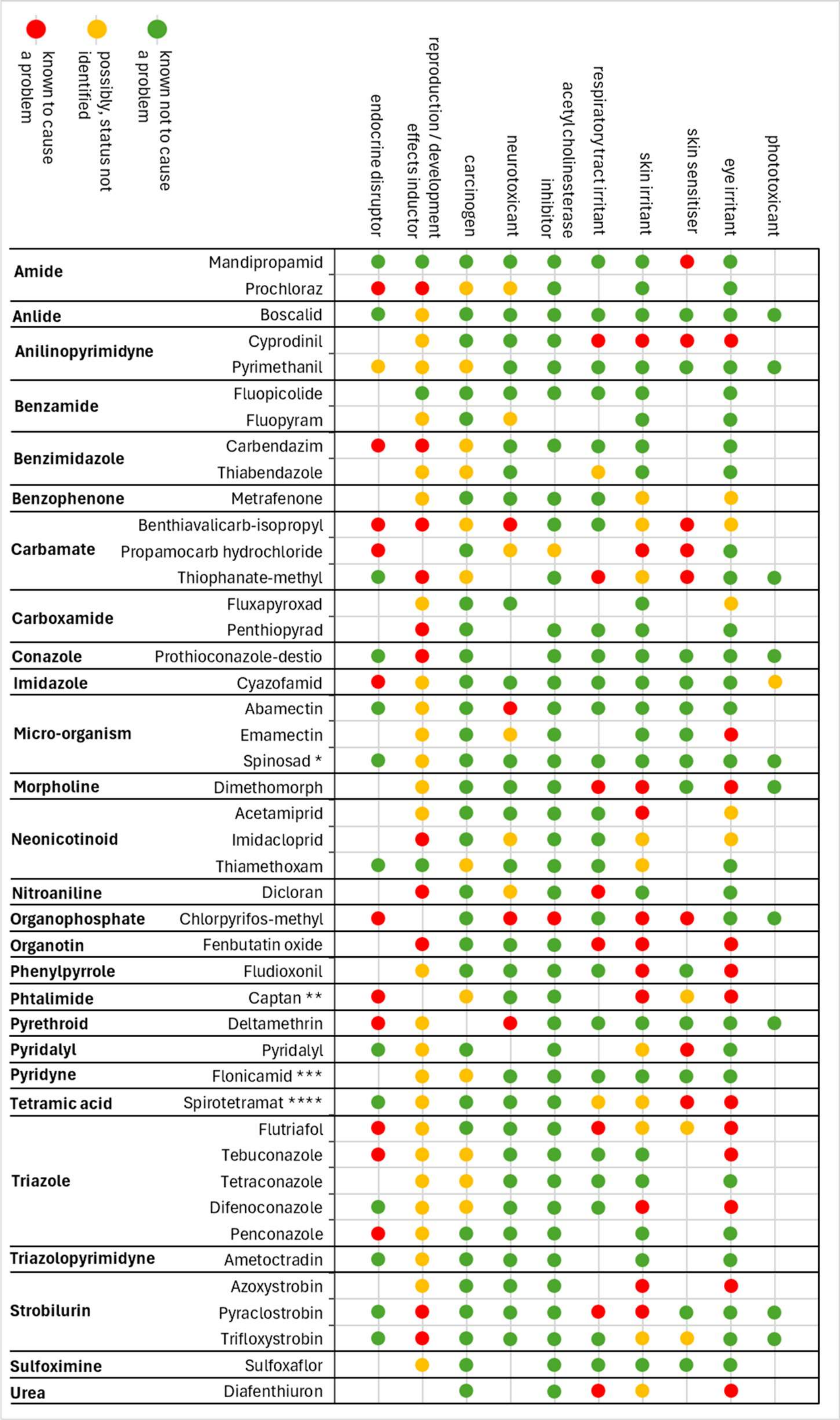

Figure S3. Detailed toxicological effects of detected pesticides in vegetable samples.

Table S1. Determined pesticides and validation parameters: recoveries (%), relative standard deviation (RSD%), correlation coefficient (R2), limits of quantification (LOQ), matrix effect (ME%) and expanded uncertainties (U%) of 552 pesticides in eggplant, avocado, chickpea, lettuce, onion, soyabean, and tomato

excel file

Table S2. Results of participation in proficiency testing

Pesticide	Assigned value [mg/kg]	Labolatory results [mg/kg]	z-score
Beans Homogenate			
EUPT-SRM 20, European Commission – National Food Institute Technical University of Denmark			
Propamocarb	0,0533	0,071	1,3
Tomato Homogenate			
EURL-PT-FV-24, European Commission - University of Almeria (Spain)			
Acetamipryd	0,053	0,049	-0,3
Azoxystrobin	0,089	0,085	-0,2
Buprofezin	0,074	0,071	-0,2
Chlorfenvinphos	0,084	0,081	-0,2
Chlorpyrifos	0,072	0,078	0,4
Deltamethrin	0,087	0,1	0,6
Diazinon	0,616	0,52	-0,6
Fenamiphos	0,057	0,058	0,0
Flonicamid	0,099	0,08	-0,8
Fluopyram	0,457	0,42	-0,3
Oxamyl	0,084	0,076	-0,4
Oxydemeton-methyl	0,098	0,082	-0,7
Procymidone	0,177	0,18	0,1
Propamocarb	0,588	0,49	-0,7
Spinosad	0,139	0,209	2,0
Zoxamide	0,046	0,043	-0,3
Tomato Homogenate			
EUPT-SRM 17, European Commission – National Food Institute Technical University of Denmark			
Captan	0.172	0.20	0.7
Chlorotalonil	0.151	0.19	1.0
Dithiocarbamates	0.187	0.17	-0.4
Dodine	0.100	0.12	0.8
Emamectin B1a	0.046	0.055	0.8
Folpet	0.249	0.29	0.7
Pymetrozine	0.150	0.095	-1.5

Table S3. Chromatographic conditions and the triple quadrupole system parameters of GC/LC/MS/MS instrument.

System LC	Eksigent Ultra LC−100	System GC	Agilent 7890B
Column	KINETEX C18 2.6 µm, 2.1 x 100 mm	Column	HP−5MS<br>30 m × 0.25 mm ID and 0.25 µm
Column temperature	40 °C	Column temperature	70 °C
Injection volume	10 µl	Injection mode	Splitless
Mobile phase	A: water with 0.5% formic acid and 2mM ammonium formate<br>B: methanol with 0.5% formic acid and 2 mM ammonium formate	Injection volume<br>Injection temperature	2 µl<br>270 °C
Gradient	0−0.5 min 1% B→0.5−5 min 1−90% B→5−7 min 90% B→7−8 min 90−1% B→8−10 min 1% B	Oven temperature	70 °C (2 min hold) → 150 °C at 25 °C/min → 200 °C at 3 °C/min → 280 °C at 8 °C/min (10 min hold).
Flow rate	0.50 mL/min	Carrier gas (flow)	Helium (2.1 mL/min)
Total running time	10 min	Total running time	42.88 min
System MS/MS	6500 QTRAP	System MS/MS	Agilent 7000B
Ionisation mode	Electrospray positive ion mode	Ionisation mode	Electron ionisation source (−70 eV)
Capillary voltage	5000 V	Transfer line temperature	280 °C
Turbo heaters	400 °C	Ion source temperature	300 °C
Nebulizer gas (pressure)	Nitrogen (60 psi)	Quadrupoles temperature	180 °C, 180 °C
Auxiliary gas (pressure)	Nitrogen (50 psi)	Collision gases (flow)	Helium (2.25 mL/min)
Curtain gas (pressure)	Nitrogen (30 psi)		Nitrogen (1.5 mL/min)

Table S4. Acquisition parameters analyzed pesticides and internal standards (ISs).

a) LC/MS/MS

No.	Pesticides	Precursor ion (m/z)	Quantification			Confirmation			DP(V)	EP(V)
			Product ion (m/z)	CE (V)	CXP (V)	Product ion (m/z)	CE (V)	CXP (V)		
1.	2, 4−D	218.9	161	20	20	220.9	20	20	65	5
2.	3−hydroxycarbofuran	238.1	181.1	17	10	163	21	10	46	10
3.	8,9−Z−avermectin B1a	890.5	305.1	23	14	567.3	23	14	20	10
4.	Acetamiprid	223	125.9	27	6	99	51	5	80	10
5.	Acibenzolar−S−methyl	211	136	39	8	91	28	11	96	10
6.	Aclonifen	265.1	248	25	14	182.1	39	10	61	10
7.	Alachlor	271.1	162.1	20	10	238.1	11	10	28	10
8.	Aldicarb	208.1	116.1	11	8	89	21	14	26	10
9.	Aldicarb sulfone	223.1	148	13	8	86	23	14	36	10
10.	Aldicarb sulfoxide	207	132	11	8	88.9	21	10	36	10
11.	Allethrin	303.2	123	25	6	91	55	4	66	10
12.	Ametoctradin	276.233	149	51	8	176.1	53	10	96	10
13.	Ametryn	228.1	186	25	10	96	33	14	86	10
14.	Amidosulfuron	370	261	19	14	218	33	12	25	10
15.	Aminocarb	209.1	137.1	33	8	152.1	19	10	56	10
16.	Amisulbrom	466	227	5	25	148.1	5	12	81	10
17.	Anilazine	275	153	37	12	178	35	10	76	10
18.	Anthraquinone	209.2	152	45	12	153.1	33	8	76	10
19.	Atrazine desisopropyl	174.1	104.1	33	18	132.1	25	8	61	10
20.	Avermectin B1a	890.4	305.1	33	16	567.2	21	26	62	10
21.	Avermectin B1b	876.5	291	35	10	145	43	6	91	10
22.	Azadirachtin	703.219	567.1	21	8	585.1	19	8	96	10
23.	Azamethiphos	325	112.1	51	2	138.9	37	8	86	10
24.	Beflubutamid	356.2	91.1	53	6	65	105	10	66	10
25.	Bendiocarb	224.11	167.1	13	10	109	23	8	36	10
26.	Benfuracarb	411.2	195.1	28	10	89.9	21	10	66	10

No.	Pesticides	Precursor ion (m/z)	Quantification			Confirmation			DP(V)	EP(V)
			Product ion (m/z)	CE (V)	CXP (V)	Product ion (m/z)	CE (V)	CXP (V)		
27.	Benomyl	291.2	160.1	39	12	132.1	69	8	41	10
28.	Bensulfuron−methyl	411	149	27	8	118.9	57	12	101	10
29.	Benthiavalicarb−isopropyl	382.1	180	41	10	116	27	6	91	10
30.	Benzoximate	364	199	13	10	104.9	35	12	56	10
31.	Bifenazate	301.1	170	27	10	151.9	55	8	76	10
32.	Bifenox	341.9	309.9	10	10	188.9	22	10	16	10
33.	Bixafen	414.2	393.9	21	24	266	33	14	76	10
34.	Bromacil	261	204.9	19	12	187.9	37	10	36	10
35.	Butafenacil	492.1	331	33	18	180	57	10	46	10
36.	Butocarboxim	207	132.1	9	8	74.9	19	12	31	10
37.	Butocarboxim sulfoxide	223.1	106	11	6	166.1	15	8	40	10
38.	Buturon	237	84	21	14	125.9	39	6	91	10
39.	BYI08330−enol	302.1	216.1	28	13	268.1	24	14	46	10
40.	BYI08330−enol−glucoside	464.2	302.1	12	13	270.1	48	15	22	10
41.	BYI08330−ketohydroxy	318.1	300.2	18	12	270.1	18	10	24	10
42.	BYI08330−monohydroxy	304.1	254.1	20	10	272.1	28	11	36	10
43.	Carbendazim	192	160.1	27	10	132.1	43	8	71	10
44.	Carbetamide	237.1	192.1	13	12	118.1	17	6	36	10
45.	Carfentrazone−ethyl	412	345.9	33	18	365.9	25	20	126	10
46.	Chlorbromuron	292.9	204	28	10	182	25	9	51	10
47.	Chlorfluazuron	540	382.9	31	20	158	25	10	81	10
48.	Chloridazon	222	104	31	12	92	33	14	11	10
49.	Chlorotoluron	213.1	72	21	10	46	37	12	76	10
50.	Chloroxuron	291	72	23	8	218	33	11	106	10
51.	Chlorsulfuron	358	167	23	10	141.1	23	6	25	10
52.	Chromafenozide	395.2	175	21	10	339.1	11	19	40	10
53.	Cinosulfuron	414	183	23	10	215	22	11	76	10
54.	Clethodim	360.1	164.1	29	10	268.1	17	16	61	10

No.	Pesticides	Precursor ion (m/z)	Quantification			Confirmation			DP(V)	EP(V)
			Product ion (m/z)	CE (V)	CXP (V)	Product ion (m/z)	CE (V)	CXP (V)		
55.	Clodinafop−propargyl	350	266	21	14	90.9	35	14	101	10
56.	Clofentezine	303	138	19	8	101.9	49	12	51	10
57.	Cloquintocet meksylowy	336.2	237.9	21	20	191.9	37	30	86	10
58.	Clothianidin	250	169	19	10	132	21	6	6	10
59.	Coumaphos	363.004	227	37	12	306.9	25	18	81	10
60.	Crimidin	172	106.9	35	14	95.1	31	8	91	10
61.	Cyanazine	241.1	214.1	25	12	104	43	18	61	10
62.	Cyantraniliprole	472.7	283.9	14	10	442.0	17	10	32	10
63.	Cyazofamid	325.1	108	19	8	261	13	14	16	10
64.	Cycloxydim	326.2	280.1	19	14	180	27	10	91	10
65.	Cycluron	199.1	89	21	10	72	29	10	71	10
66.	Cymoxanil	199	128	13	4	111	25	4	81	10
67.	Cyprazine	228.1	186	25	10	108	33	12	96	10
68.	Dazomet	163.1	107.2	11	6	89.1	15	14	51	10
69.	Demeton−S	259	88.9	21	14	61	49	10	21	10
70.	Demeton−S−methyl	231	88.9	17	12	61	41	10	21	10
71.	Demeton−S−methyl−sulfone	263	169	21	12	108.9	37	14	76	10
72.	Desmedipham	318.1	182.1	19	10	136	37	6	16	10
73.	Diafenthiuron	385.1	329.1	27	16	278.1	43	14	121	10
74.	Diclobutrazol	328	69.9	58	8	159	48	8	85	10
75.	Didecyldimethylammonium chloride (DDAC)	326.2	186.2	39	12	41.1	93	6	61	10
76.	Diethofencarb	268.1	226.1	13	12	180	25	10	26	10
77.	Difenoxuron	287	123	25	8	71.9	23	10	121	10
78.	Diflubenzuron	311.1	158.1	19	10	141	47	8	61	10
79.	N,N−dimethyl−N'−p−tolysulphamide (DMST)	215.2	106	19	8	77.1	55	14	41	10
80.	Dimefuron	339	166.9	29	10	256	23	14	141	10
81.	Dimethylaminosulfanilide (DMSA)	201.2	137.2	13	24	91.9	27	6	46	10
82.	Dinotefuran	203	157.1	11	10	113.1	17	8	36	10

No.	Pesticides	Precursor ion (m/z)	Quantification			Confirmation			DP(V)	EP(V)
			Product ion (m/z)	CE (V)	CXP (V)	Product ion (m/z)	CE (V)	CXP (V)		
83.	Dioxacarb	224	167.1	13	10	123	23	6	41	10
84.	Disulfoton	275	89	20	10	61	46	10	56	10
85.	Disulfoton−sulfon	307	96.8	41	12	171	16	9	66	10
86.	Disulfoton sulfoxide	291.1	185	19	12	212.9	15	12	46	10
87.	Diuron	233	72	23	8	46.1	39	12	86	10
88.	Dodine	228.3	57	41	10	186.2	27	10	91	10
89.	Doramectin	916.4	331.1	33	16	593.2	21	28	81	10
90.	Emamectin B1a	886.4	158.1	41	10	126	83	22	121	10
	Emamectin B1b	872.4	158.1	43	8	126.2	79	16	106	10
91.	Eprinomectin B1a	914.4	186.1	23	4	112	95	6	160	10
92.	Ethametsulfuron−methyl	411.2	196.1	15	10	168.1	30	10	46	10
93.	Ethiofencarb	226.1	107.1	23	8	164.1	11	10	41	10
94.	Ethiofencarb−sulfone	258	107.1	21	8	200.9	11	10	51	10
95.	Ethiofencarb−sulfoxide	242	106.9	23	12	185	13	8	41	10
96.	Ethiprole	397	350.8	29	18	254.9	47	14	116	10
97.	Ethirimol	210.1	140.1	29	8	97.9	35	12	116	10
98.	Ethoxyquin	218.2	148.0	31	12	174.1	41	14	61	10
99.	Ethoxysulfuron	399	261	25	14	218	35	14	56	10
100	Fenamiphos sulfone	336.1	265.9	29	14	308	23	18	81	10
101	Fenamiphos sulfoxide	320.2	171	33	10	233	35	12	86	10
102	Fenbutatin oxide	519.1	90.9	103	14	463.1	33	22	171	10
103	Fenchlorazole−ethyl	403.9	357.8	31	18	375.7	21	20	116	10
104	Fenfuram	202	109	27	8	120	21	6	91	10
105	Fenhexamid	302.1	97.1	31	6	55	57	8	86	10
106	Fenobucarb	208.1	95.1	21	8	152.1	11	10	36	10
107	Fenpicoxamid	615	124,1	74	10	239	22	10	35	10
108	Fenpyroximate	422.1	366.1	23	18	135	41	8	116	10
109	Fensulfothion sulfone	325	268.9	21	14	296.9	15	18	101	10
110	Fenthion sulfone	311.1	125	29	8	278.8	27	16	76	10

No.	Pesticides	Precursor ion (m/z)	Quantification			Confirmation			DP(V)	EP(V)
			Product ion (m/z)	CE (V)	CXP (V)	Product ion (m/z)	CE (V)	CXP (V)		
111	Fenthion sulfoxide	295.1	279.7	25	16	108.9	45	18	76	10
112	Fenthion oxon sulfone	295	104.1	35	6	217.1	27	12	96	10
113	Fenthion oxon sulfoxide	279.1	104.1	39	18	264.1	27	16	56	10
114	Fenthion oxon	263.1	216	33	14	231	23	14	76	10
115	Fenuron	165.1	72	21	10	46.1	19	8	91	10
116	Fipronil sulfone	451	415	26	38	282	10	38	28	10
117	Flazasulfuron	408	182	25	10	227	28	17	66	10
118	Florasulam	359.9	128.9	20	20	81.6	20	20	50	10
119	Florpyrauxifen−benzyl	439	91	63	5	65	113	10	30	10
120	Fluazifop	328	282	27	16	254	35	14	91	10
121	Fluazinam	463	451.9	30	28	397.9	21	26	30	10
122	Fluazuron	506	158	27	10	141	67	8	126	10
123	Flubendiamid	700	408	19	20	273.9	47	14	50	10
124	Fluconazole	307.1	238,2	18	14	169	34	9	80	10
125	Fluensulfone	292	166	25	10	109	25	10	40	10
126	Flufenoxuron	489	158	25	10	141	71	8	111	10
127	Flumethrin	510.2	239	29	14	267	17	16	91	10
128	Flumioxazin	355	327.2	20	18	299	38	16	136	10
129	Fluometuron	233.1	72.1	23	8	145.1	47	8	86	10
130	Fluopyram	397	173	39	12	145	43	14	54	10
131	Flupyradifurone	288.9	126	5	33	245	5	23	76	10
132	Fluoroglycofene−ethyl	465	343.8	19	18	222.9	45	12	51	10
133	Fluxapyroxad	382.083	342.1	29	20	362.2	21	4	71	10
134	Fluoxastrobin	459	427	25	22	188	45	10	101	10
135	Fluridone	330	310	39	16	259	63	14	161	10
136	Fluthiacet−methyl	404	273.9	39	16	215	53	12	151	10
137	FM−6−1	294.944	215	31	12	43.2	53	2	71	10
138	Foramsulfuron	453	182.2	35	12	272	21	16	71	10

No.	Pesticides	Precursor ion (m/z)	Quantification			Confirmation			DP(V)	EP(V)
			Product ion (m/z)	CE (V)	CXP (V)	Product ion (m/z)	CE (V)	CXP (V)		
139	Forchlorfenuron	248	129	23	6	93	47	10	36	10
140	Formetanate	222	165.1	21	10	120.1	35	7	16	10
141	Furalaxyl	302.1	242.1	21	12	95	33	8	66	10
142	Furathiocarb	383.1	195	25	12	252	17	14	86	10
143	Halauxifen−methyl	346	286	5	22	251	5	32	30	10
144	Halofenozide	331	105	23	6	275	15	12	60	10
145	Halosulfuron−methyl	435	182	31	10	83.1	79	4	61	10
146	Haloxyfop	360	288	20	20	290	20	20	40	5
147	Haloxyfop−ethoxyethyl	434	315.9	27	16	90.9	45	14	106	10
148	Hexazinone	253.1	171	23	10	71	41	12	66	10
149	Hexaflumuron	461	158.1	23	10	141	63	8	111	10
150	Hydramethylnon	495.1	323.1	43	18	151	95	8	211	10
151	Imazamox	306.2	261.1	31	14	193.1	37	12	66	10
152	Imidacloprid	256	209.1	21	12	175.1	27	10	80	10
153	Iodosulfuron−methyl	507.9	167	25	10	141	40	8	71	10
154	Isofetamid	360,1	125	15	10	182	10	10	20	10
155	Isoproturon	207.1	72	23	10	46.1	37	10	91	10
156	Isopyrazam	360.116	320.1	29	16	340.1	23	4	81	10
157	Isoxaben	333.1	165	25	10	106.9	81	12	71	10
158	Isoxadifen−ethyl	313.2	232.1	27	12	204.2	39	12	34	10
159	Isoxaflutole	377.1	69	19	14	251	27	14	55	10
160	Isoxaflutole diketonitrile	377	250.9	33	14	69	31	2	51	10
161	Ivermectin	892.4	307.1	32	14	569.3	21	26	94	10
162	Linuron	249	160	25	10	182	21	10	61	10
163	Lufenuron	511	158.1	25	10	140.9	73	8	111	10
164	Mandestrobin	314.4	192	5	7	160	5	17	20	10
165	Mefenacet	299.1	148	19	8	120.1	35	8	51	10
166	Mefenpyr−diethyl	373.1	326.9	23	16	160.1	47	10	70	10

No.	Pesticides	Precursor ion (m/z)	Quantification			Confirmation			DP(V)	EP(V)
			Product ion (m/z)	CE (V)	CXP (V)	Product ion (m/z)	CE (V)	CXP (V)		
167	Mefentrifluconazole	397.8	70	25	10	181.9	30	10	20	10
168	Mendipropamid	412.1	328	21	18	356	15	18	96	10
169	Mepanipyrim	224	106	33	18	66	59	10	131	10
170	Mepronil	270.1	119.1	31	8	228	20	12	91	10
171	Mesosulfuron−methyl	504	182	29	10	83	81	10	101	10
172	Mesotrione	357	340.1	12	18	228.1	28	12	30	10
173	Metaflumizon	507.1	178	33	10	287	35	16	151	10
174	Methabenzthiazuron	222.1	165	23	10	150	43	8	46	10
175	Methfuroxam	230	137	27	8	111.1	21	8	81	10
176	Methiocarb	226.1	169	13	10	121	25	6	41	10
177	Methiocarb−sulfon	258	122	25	6	201.1	13	4	81	10
178	Methiocarb−sulfoxid	242	185	19	10	122.1	39	8	56	10
179	Methomyl	163	88	13	10	105.9	13	12	6	10
180	Methoprotryne	272.1	198.1	31	12	240.1	25	12	81	10
181	Methoxyfenozide	369.1	149	23	8	133	34	7	46	10
182	Metobromuron	259	169.9	25	10	148	21	8	66	10
183	Metolcarb	166	109.1	15	8	94	41	14	26	10
184	Metosulam	418	175	33	10	140	71	8	106	10
185	Metoxuron	229	72	21	8	46.1	37	8	76	10
186	Metsulfuron−methyl	382	167	21	10	198.9	29	12	61	10
187	Mexacarbate	223.1	166	19	8	151.1	31	8	21	10
188	Monocrotophos	224	127	23	6	98.1	17	8	41	10
189	Monolinuron	215	125.9	23	6	99	45	12	61	10
190	Monuron	199.1	71.9	21	10	126	33	6	76	10
191	Moxidectin	640.3	528.2	13	26	199.1	33	10	80	10
192	Naled	378.7	127	16	6.5	109	36	6.5	24	10
193	Neburon	275	88.1	21	10	114.1	21	8	101	10
194	Nicosulfuron	411.1	182	27	10	213	23	12	86	10

No.	Pesticides	Precursor ion (m/z)	Quantification			Confirmation			DP(V)	EP(V)
			Product ion (m/z)	CE (V)	CXP (V)	Product ion (m/z)	CE (V)	CXP (V)		
195	Nitenpyram	271.1	126	37	8	237	25	11	61	10
196	Norflurazon	304	283.9	33	16	160	43	10	131	10
197	Novaluron	493	158.1	25	10	141	65	8	96	10
198	Omethoate	214	182.9	15	10	124.9	29	6	46	10
199	Oxadiargyl	341.1	223	20	10	150.9	26	15	50	10
200	Oxadixyl	279.1	219.1	15	12	133.1	29	8	46	10
201	Oxamyl	237	72	31	8	90	11	8	25	10
202	Oxamyl oxime	163	72.1	17	6	90	23	14	46	10
203	Oxathiapiprolin	540.1	162.9	50	10	499.9	50	10	48	10
204	Oxycarboxin	268	174.9	21	10	147	31	10	66	10
205	Oxydemeton−methyl	247	169	19	10	124.9	29	6	41	10
206	Pencycuron	329	124.9	29	6	218	31	6	70	10
207	Penflufen	318.124	234.1	23	12	141	43	8	76	10
208	Penthiopyrad	360.049	276	21	14	176.9	43	10	71	10
209	Penoxsulam	484	195	5	30	164	5	30	10	10
210	Petoxamid	296.1	131.1	27	6	250	17	14	11	10
211	Phenmedipham	301.1	168.1	13	10	136	29	8	76	10
212	Phorate−sulfoxide	277	198.9	13	12	142.9	27	8	31	10
213	Phorate sulfone	293	171	15	8	96.8	47	14	51	10
214	Phosmet oxon	302.1	160.1	21	8	133.1	51	6	61	10
215	Phosphamidon	300.2	174	21	10	127.1	29	12	66	10
216	Phoxim	299	77	43	12	129	15	6	46	10
217	Picolinafen	377	238	37	12	359	27	18	106	10
218	Pinoxaden	401.3	317.1	53	4	57.1	29	18	91	10
219	Piperonyl butoxide	356.2	177	17	10	119.1	47	8	26	10
220	Pirimicarb−desmethyl	225.1	72	33	12	168	19	10	56	10
221	Prallethrin	301.3	105.1	31	6	123	23	8	66	10
222	Primisulfuron−methyl	469	254	29	14	199	31	10	76	10

No.	Pesticides	Precursor ion (m/z)	Quantification			Confirmation			DP(V)	EP(V)
			Product ion (m/z)	CE (V)	CXP (V)	Product ion (m/z)	CE (V)	CXP (V)		
223	Procyazine	253	226	23	15	186	20	11	40	10
224	Profoxydim	468.1	280.1	23	14	106.9	81	12	106	10
225	Promecarb	208.1	151	13	8	109.1	23	8	26	10
226	Prometon	226.1	142	31	8	184.1	25	10	81	10
227	Prometryn	242.09	158	31	10	200.1	25	12	76	10
228	Propamocarb hydrochloride	189.2	102.1	25	8	74	35	12	61	10
229	Propoxycarbazone−sodium	421.2	180.1	21	10	138.1	39	8	61	10
230	Proquinazid	373	288.9	33	16	330.9	19	18	71	10
231	Prosulfuron	420	141	25	8	167	25	10	76	10
232	Prothioconazole−desthio	312	69.9	61	8	125	37	6	96	10
233	Pymetrozine	218.1	104.9	27	12	78	59	12	81	10
234	Pyracarbolid	218.1	125	25	6	97	36	6	21	10
235	Pyraflufen−ethyl	413	338.9	5	17	289	5	30	30	10
236	Pyrethrins	317.2	149.0	15	8	107.1	27	6	61	10
237	Pyridafol	207	77	30	10	103.9	22	10	46	10
238	Pyridalil	489.9	108.9	29	6	183	26	10	101	10
239	Pyridate	379.1	206.9	23	12	350.9	15	20	51	10
240	Pyriofenone	365.8	184.0	30	12	209	33	14	40	10
241	Pyroxsulam	435	194.9	5	35	257.8	5	35	25	10
242	Quinclorac	242,0	223,9	21	14	161	53	10	41	10
243	Quinmerac	222.1	203.9	21	14	206	23	12	41	10
244	Quinoclamine	222.1	203.9	21	14	206	23	12	36	10
245	Rimsulfuron	432	182	29	10	325	21	18	76	10
246	Rotenone	395.1	213	31	12	192.1	33	10	136	10
247	Secbumeton	226.1	170.1	25	10	100	39	12	81	10
248	Sedaxane	332.1	159	5	31	139	5	33	56	10
249	Siduron	233.1	137	23	8	94.1	29	8	96	10
250	Silthiofam	268	139	26	14	73	45	14	122	10

No.	Pesticides	Precursor ion (m/z)	Quantification			Confirmation			DP(V)	EP(V)
			Product ion (m/z)	CE (V)	CXP (V)	Product ion (m/z)	CE (V)	CXP (V)		
251	Simetryn	214.1	124.1	27	8	144	28	7	86	10
252	Spinetoram J	748.9	142.1	29	10	97.9	60	10	44	10
	Spinetoram L	760.8	142.1	28	10	98.2	65	10	44	10
253	Spinosad Spinosyn A	732.4	142.1	35	8	98	103	16	136	10
	Spinosyn D	746.4	142	35	8	98.1	101	16	146	10
254	Spirotetramat	374.1	302	23	16	330.1	21	20	131	10
255	Sulcotrione	329	139.1	18	10	69.2	38	10	36	10
256	Sulfoxaflor	275.9	212.9	22	9	211.7	22	11	70	10
257	Sulfentrazone	387	306.9	29	16	145.9	55	8	146	10
258	Sulfometuron−methyl	365	150	23	8	107	59	12	66	10
259	Sulfosulfuron	471	211	19	12	261	23	12	76	10
260	Tebufenozide	353.1	133.1	25	6	297.1	11	16	41	10
261	Tebuthiuron	229.08	172.1	25	10	116	35	8	81	10
262	Teflubenzuron	381	158	21	10	141	49	8	81	10
263	Tembotrione	458	340.9	23	20	262	53	14	56	10
264	Tepraloxydim	342.11	250.1	19	14	166	29	10	86	10
265	Terbumeton	226	170	25	10	114.1	33	8	81	10
266	Terbutryne	242.1	186.1	25	10	91	35	10	76	10
267	TFNA	192	79	51	4	98	43	4	76	10
268	TFNG	249	203	27	12	98	61	4	76	10
269	Thiabendazol	202	175	37	10	131.1	45	8	121	10
270	Thiacloprid	253	126	29	6	72.9	81	8	96	10
271	Thiamethoxam	292	211	17	12	181	31	10	61	10
272	Thidiazuron	221.1	102	21	12	127.9	23	12	61	10
273	Thiencarbazone−methyl	391.2	359	5	15	130	5	35	71	10
274	Thifensulfuron−methyl	388	167	21	8	204.9	35	12	61	10
275	Thiobencarb	258.1	125	25	6	89	69	10	36	10
276	Thiodicarb	355	88	27	10	108	21	8	51	10
277	Thiofanox−sulfone	251.1	57.2	15	10	75.9	15	10	50	10

No.	Pesticides	Precursor ion (m/z)	Quantification			Confirmation			DP(V)	EP(V)
			Product ion (m/z)	CE (V)	CXP (V)	Product ion (m/z)	CE (V)	CXP (V)		
278	Thiofanox−sulfoxide	252.1	104	16	9	57	35	8	50	10
279	Thiophanate−ethyl	371	150.9	27	8	325	17	17	71	10
280	Thiophanate−methyl	343	151	27	8	192	21	10	81	10
281	Topramezone	364	334	16	10	125	29	10	31	10
282	Tralkoxydim E	330.1	284.1	17	14	138	27	6	76	10
283	Tralkoxydim Z	330.1	284.1	17	14	138	27	6	76	10
284	Transfluthrin	388.2	163	41	8	163.2	37	8	36	10
285	Triasulfuron	402	167.1	23	10	141	27	8	81	10
286	Triazoxide	248	95	36	7	68	35	8	141	10
287	Tribenuron−methyl	396	155	19	8	181	27	10	76	10
288	Trichlorfon	257	108.9	27	18	220.8	17	14	61	10
289	Tricyclazole	190	163	31	10	136	39	8	96	10
290	Tridemorf	298	130	23	10	98	23	10	10	10
291	Triflumuron	359	156	21	10	139	45	8	56	10
292	Triflusulfuron−methyl	493	264	29	14	238	35	12	76	10
293	Triforine	434.91	389.8	17	20	98	43	14	60	10
294	Trinexapac−ethyl	253.1	207.1	19	12	68.9	33	12	80	10
295	Tritosulfuron	446	195	18	10	145	34	10	36	10
296	Uniconazole	292.1	70	59	10	125	37	8	106	10
297	Vamidothion	288	146	17	8	117.9	33	9	36	10
298	Valifenalate	399.2	155	5	47	161.1	5	33	76	10
299	Atrazine − d5 (IS)	221.1	179.1	25	10	69	101	10	51	10
300	Carbendazim − d3 (IS)	195	160	25	10	132	20	10	66	10
301	Izoproturon − d6 (IS)	213.1	78.3	27	14	171.2	21	10	66	10

CE−collision energy, CXP−cell exit potential , DP−declustering potential, EP−entrance potential

b) GC/MS/MS

No.	Active substance	MRM transitions m/z (collision energy eV)		
		**Quantitative ion pairs**	**Qualitative ion pairs I**	**Qualitative ion pairs II**
1.	2,6−dichlorobenzamide	173 >145 (15)	189 > 173 (5)	173 > 109 (30)
2.	2−phenylphenol	169 > 141 (15)	169 > 115 (25)	170 > 141 (25)
3.	Acephate	136 > 94 (10)	125 > 47 (15)	142 > 95 (5)
4.	Acetochlor	174 > 146 (10)	146 > 131 (10)	223 > 132 (20)
5.	Acrinathrin	207 > 181 (10)	181 > 152 (30)	289 > 93 (10)
6.	Aldrin	263 > 193 (35)	255 > 220 (20)	263 > 191 (35)
7.	Amitraz	132 > 117 (15)	162 > 132 (5)	162 > 121 (10)
8.	Atrazine	215 > 58 (10)	215 > 200 (5)	200 > 122 (5)
9.	Atrazine−desethyl	186>172 (5)	172>94 (15)	172>69 (20)
10.	Azaconazole	217 > 173 (15)	219 > 175 (15)	173 > 145 (15)
11.	Azinphos − ethyl	132 > 77 (15)	160 > 77 (20)	160 > 132 (10)
12.	Azinphos − methyl	160 > 132 (10)	132 > 77 (15)	160 > 77 (20)
13.	Azoxystrobin	344 > 329 (15)	344 > 172 (40)	344 > 183 (25)
14.	Benalaxyl	266 > 148 (5)	148 > 105 (20)	148 > 77 (35)
15.	Benfluralin	292>264 (5)	292>206 (10)	263>206 (10)
16.	Beta cyfluthrin	163 > 91 (15)	163 > 127 (5)	199 > 170 (25)
17.	Bifenthrin	181 > 166 (10)	181 > 165 (25)	166 > 165 (20)
18.	Biphenyl	154>153 (15)	155>154 (15)	153.1>152 (15)
19.	Bitertanol	170 > 141 (20)	170 > 115 (40)	168 > 70 (10)
20.	Boscalid	140 > 76 (15)	140 > 112 (10)	112 > 76 (15)
21.	Bromophos − ethyl	359 > 303 (15)	303 > 285 (15)	242 > 97 (30)
22.	Bromophos − methyl	125 > 47 (10)	331 > 316 (15)	125 > 79 (5)
23.	Bromopropylate	183 > 155 (15)	185 > 157 (15)	339 > 183 (20)
24.	Bromuconazole	173 > 145 (15)	173 > 109 (30)	175 > 147 (15)
25.	Bupirimate	273 > 193 (5)	273 > 108 (15)	208 > 165 (10)
26.	Buprofezin	105 > 104 (10)	105 > 77 (20)	119 > 91 (15)
27.	Butralin	266>220 (10)	266>174 (20)	224>132 (15)
28.	Cadusafos	159 > 97 (15)	159 > 131 (5)	158 > 97 (15)
29.	Captafol	150 > 79 (5)	183 > 79 (10)	150 > 72 (5)
30.	Captan	151 > 80 (5)	151 > 79 (15)	149 > 79 (10)
31.	Carbaryl	144 > 115 (20)	144 > 116 (10)	116 > 115 (10)
32.	Carbofuran	164 > 149 (10)	149 > 121 (5)	149 > 77 (30)
33.	Carbosulfan	164 > 149 (10)	118 > 76 (5)	164 > 103 (25)

No.	Active substance	MRM transitions m/z (collision energy eV)		
		Quantitative ion pairs	Qualitative ion pairs I	Qualitative ion pairs II
34.	Carboxin	235 > 143 (10)	235 >87 (20)	143 > 87 (5)
35.	Chinomethionate	233>206 (10)	233>148 (15)	206>148 (25)
36.	Chlorantraniliprole	239 > 214 (20)	277 > 243 (10)	277 >250 (10)
37.	Chlordan cis	271>236 (15)	372>265 (15)	374>265 (15)
38.	Chlordan trans	271>236 (15)	372>265 (15)	374>265 (15)
39.	Chlorfenapyr	136>102 (15)	246>227 (15)	327>246 (15)
40.	Chlorfenson	175>111 (10)	111>75 (15)	177>113 (10)
41.	Chlorfenvinphos	267 > 159 (15)	323 > 267 (10)	269 > 161 (15)
42.	Chlorobenzilate	139>111 (10)	251>139 (15)	139>75 (30)
43.	Chlorothalonil	264 > 168 (25)	264 > 229 (20)	266 > 231 (20)
44.	Chlorpropham	153 > 125 (10)	153 > 90 (25)	127 > 65 (25)
45.	Chlorpyrifos	314 > 258 (15)	199 > 171 (15)	197 > 169 (15)
46.	Chlorpyrifos − methyl	125 > 47 (15)	125 > 79 (5)	286 > 93 (20)
47.	Chlorthal−dimethyl	298>221 (25)	300>223 (25)	331>300 (10)
48.	Chlozolinate	186>154 (15)	188>147 (15)	186>109 (30)
49.	Clomazone	125 > 89 (15)	204 > 107 (20)	125 > 99 (15)
50.	Cyflufenamid	118 > 90 (10)	118 > 89 (25)	188 > 88 (35)
51.	Cyfluthrin	163 > 91 (15)	163 > 127 (5)	199 > 170 (25)
52.	Cypermethrin	163 > 127 (5)	163 > 91 (10)	165 > 91 (10)
53.	Cyphenothrin	123>81 (5)	181>152 (25)	208>141 (15)
54.	Cyproconazole	139 > 111 (15)	222 > 125 (15)	222 > 82 (10)
55.	Cyprodinil	225 > 224 (10)	224 > 208 (20)	226 > 225 (10)
56.	Deltamethrin	253 > 93 (15)	181 > 152 (25)	251 > 172 (5)
57.	Diazinon	199 > 93 (15)	137 > 84 (10)	137 > 54 (20)
58.	Dichlobenil	171 > 100 (25)	171 > 136 (15)	173 > 100 (25)
59.	Dichlofluanid	123 > 77 (20)	224 > 123 (10)	226 > 123 (10)
60.	Dichlorvos	109 > 79 (5)	185 > 93 (10)	145 > 109 (10)
61.	Diclofop−methyl	339>252 (10)	280>119 (10)	253>162 (15)
62.	Dicloran	206 > 176 (10)	160 > 124 (10)	124 > 73 (10)
63.	Dicofol	250 > 139 (20)	139 > 111 (20)	139 > 75 (20)
64.	Dicrotophos	127 > 109 (15)	127 > 95 (15)	193 > 127 (5)
65.	Dieldrin	277 > 241 (5)	263 > 193 (35)	263 > 191 (35)
66.	Diethyltoluamide (DEET)	119 > 91 (10)	119 > 65 (20)	91 > 65 (10)
67.	Difenoconazole	323 > 265 (15)	265 > 202 (20)	325 > 267 (15)
68.	Diflufenican (DFF)	266 > 238 (15)	266 > 246 (15)	394 > 266 (10)

No.	Active substance	MRM transitions m/z (collision energy eV)		
		**Quantitative ion pairs**	**Qualitative ion pairs I**	**Qualitative ion pairs II**
69.	Dimethachlor	134 > 105 (10)	134 > 77 (25)	197 > 148 (10)
70.	Dimethenamid	230 > 154 (10)	154 > 111 (10)	232 > 154 (10)
71.	Dimethoate	86 > 46 (15)	93 > 63 (10)	87 > 86 (5)
72.	Dimethomorph	301 > 165 (10)	303 > 165 (10)	387 > 301 (10)
73.	Dimoxystrobin	205 > 116 (10)	116 > 89 (15)	116 > 63 (30)
74.	Dinikonazol	268 > 232 (10)	270 > 232 (10)	270 > 234 (10)
75.	Diphenylamine	169 > 168 (15)	168 > 167 (15)	167 > 166 (20)
76.	Dodemorph	169 > 168 (15)	168 > 167 (15)	167 > 166 (20)
77.	Endosulfan−sulphate	272 > 237 (15)	274 > 239 (15)	274 > 237 (15)
78.	Endrin	263 > 193 (35)	245 > 173 (30)	317 > 281 (5)
79.	EPN	169 > 141 (5)	169 > 77 (25)	185 > 157 (5)
80.	Epoxiconazole	192 > 138 (10)	192 > 111 (25)	165 > 138 (10)
81.	Esfenvalerate	167 > 125 (5)	209 > 141 (15)	181 > 152 (20)
82.	Etaconazole	173 > 145 (15)	173 > 109 (30)	245 > 55 (10)
83.	Ethion	153 > 97 (10)	125 > 97 (10)	231 > 175 (10)
84.	Ethofumesate	207 > 161 (5)	207 > 137 (10)	161 > 105 (10)
85.	Ethoprophos	158 > 97 (15)	158 > 114 (5)	139 > 97 (5)
86.	Etofenprox	163 > 135 (10)	163 > 107 (20)	135 > 107 (10)
87.	Etoxazole	141 > 113 (15)	141 > 63 (30)	204 > 176 (10)
88.	Famoxsadone	197 > 141 (15)	224 > 196 (10)	197 > 115 (30)
89.	Fenamidon	238 > 237 (10)	268 > 180 (20)	238 > 103 (15)
90.	Fenamiphos	154 > 139 (10)	217 > 202 (10)	303 > 288 (10)
91.	Fenarimol	219 > 107 (10)	251 > 139 (10)	139 > 75 (30)
92.	Fenazaquin	145 > 117 (10)	160 > 145 (5)	160 > 117 (20)
93.	Fenbuconazole	129 > 102 (15)	198 > 129 (5)	129 > 78 (20)
94.	Fenchlorphos	285 > 270 (15)	288 > 272 (15)	125 > 47 (15)
95.	Fenitrothion	125 > 47 (15)	125 > 79 (5)	277 > 260 (5)
96.	Fenoxaprop−P−ethyl	361 > 288 (10)	288 > 91 (20)	288 > 119 (10)
97.	Fenoxycarb	255 > 186 (10)	186 > 158 (5)	185 > 129 (5)
98.	Fenpropathrin	181 > 152 (25)	208 > 181 (5)	125 > 55 (10)
99.	Fenpropidin	98 > 55 (15)	98 > 70 (10)	117 > 91 (15)
100.	Fenpropimorph	128 > 70 (10)	128 > 110 (5)	128 > 86 (10)
101.	Fenpyrazamine	331>230 (5)	230>132 (10)	−
102.	Fenthion	278 > 109 (15)	125 > 47 (10)	124.9 > 79 (5)
103.	Fenvalerate	167>125 (5)	208>141 (15)	181>152 (20)

No.	Active substance	MRM transitions m/z (collision energy eV)		
		**Quantitative ion pairs**	**Qualitative ion pairs I**	**Qualitative ion pairs II**
104.	Fipronil	351 > 255 (15)	367 > 213 (25)	255 > 228 (15)
105.	Flonicamid	174 > 146 (10)	174 > 126 (30)	146 > 69 (30)
106.	Fluazifop−p−butyl	282 > 91 (20)	282 > 238 (20)	383 > 282 (10)
107.	Fludioxonil	248 > 127 (30)	248 > 182 (10)	248 > 154 (20)
108.	Flufenacet	151 > 136 (10)	151 > 95 (30)	123 > 95 (20)
109.	Flumetralin	175>111 (10)	111>75 (15)	177>113 (10)
110.	Fluopicolide	209 > 182 (10)	209 > 146 (20)	347 > 172 (20)
111.	Fluquinconazole	340 > 298 (15)	108 > 57 (15)	340 > 108 (40)
112.	Flurochloridone	187 > 159 (10)	311 > 174 (15)	145 > 95 (15)
113.	Fluroxypyr−1−methylheptyl	209 > 181 (10)	237 >181 (15)	237 > 209 (5)
114.	Flurtamone	333 > 120 (15)	199 > 157 (20)	157 > 137 (15)
115.	Flusilazole	233 > 165 (15)	233 > 91 (20)	315 > 233 (10)
116.	Flutolanil	173 > 145 (15)	281 > 173 (10)	173 > 95 (30)
117.	Flutriafol	123 > 95 (15)	123 > 75 (25)	219 > 123 (15)
118.	Folpet	260 > 130 (15)	262 > 130 (15)	260 > 232 (5)
119.	Fonofos	136>109 (5)	108>80 (5)	108>62 (15)
120.	Formothion	170 > 93 (5)	125 > 47 (15)	125 > 79 (5)
121.	Fosthiazate	195 > 103 (5)	195 > 60 (20)	199 > 102 (5)
122.	Fuberidazole	184 > 156 (10)	184 > 155 (30)	183 > 155 (10)
123.	Haloxyfop−methyl	316 > 91 (20)	316 > 272 (20)	375 > 316 (10)
124.	Heptachlor	100 > 58 (10)	100 > 72 (5)	198 > 126 (5)
125.	Heptachlor endo− epoxide	183 > 155 (15)	183 > 119 (25)	217 > 182 (20)
126.	Heptenophos	124 > 89 (10)	124 > 63 (35)	109 > 79 (5)
127.	Hexachlorobenzene (HCB)	284 > 214 (30)	284 > 249 (15)	289 > 212 (30)
128.	Hexaconazole	256 > 82 (10)	231 > 175 (10)	256 > 159 (15)
129.	Hexythiazox	227 > 149 (10)	184 > 149 (10)	184 > 115 (20)
130.	Imazalil	215 > 173 (5)	217 > 175 (5)	173 > 145 (15)
131.	Imibenconazole	125 > 89 (20)	125 > 99 (20)	253 > 82 (5)
132.	Indoxacarb	203 > 134 (15)	203 > 106 (25)	203 > 78 (30)
133.	Ipconazole	167 > 152 (5)	167 > 125 (5)	249 > 125 (15)
134.	Iprodione	244 > 187 (5)	187 > 124 (25)	314 > 56 (20)
135.	Iprovalicarb	119 > 91 (15)	116 > 98 (5)	116 > 55 (15)
136.	Isocarbophos	120 > 92 (10)	136 > 108 (15)	121 > 65 (15)
137.	Isofenphos	213 > 121 (10)	213 > 185 (5)	185 > 121 (5)
138.	Isofenphos − methyl	199 > 121 (10)	199 > 167 (10)	167 > 123 (5)

No.	Active substance	MRM transitions m/z (collision energy eV)		
		Quantitative ion pairs	Qualitative ion pairs I	Qualitative ion pairs II
139.	Isoprocarb	121 > 77(20)	136 > 121 (10)	121 > 103 (10)
140.	Isoprothiolane	162 > 85 (20)	162 > 134 (5)	231 > 189 (10)
141.	Krezoxim−methyl	116 > 89 (15)	116 > 63 (30)	131 > 89 (30)
142.	Lenacil	153 > 136 (15)	153 > 82 (20)	153 > 110 (15)
143.	Malaoxon	127 > 99 (5)	127 > 55 (5)	99 > 71 (5)
144.	Malathion	127 > 99 (5)	173 > 99 (15)	158 > 125 (5)
145.	Mecarbam	159 > 131 (5)	131 > 74 (5)	131 > 86 (10)
146.	Metalaxyl	234 > 146 (20)	220 > 192 (5)	234 > 174 (10)
147.	Metamitron	104 > 77 (15)	202 > 174 (5)	104 > 51 (35)
148.	Metazachlor	133 > 132 (10)	132 > 117 (15)	209 > 132 (15)
149.	Metconazole	125 > 89 (20)	125 > 99 (20)	138 > 69 (10)
150.	Methacrifos	208 > 180 (5)	125 > 47 (10)	125 > 79 (5)
151.	Methamidophos	141 > 95 (5)	95 > 79 (10)	95 > 64 (10)
152.	Methidathion	145 > 85 (5)	145 > 58 (15)	85 > 58 (5)
153.	Methoxychlor (DMDT)	227 > 169 (25)	227 > 141 (40)	227 > 212 (15)
154.	Metolachlor	238 > 162 (10)	162 > 133 (15)	240 > 162 (10)
155.	Metrafenone	209 > 166 (10)	395 > 365 (15)	227 > 169 (10)
156.	Metribuzin	198 > 82 (15)	198 > 55 (30)	144 > 128 (10
157.	Mevinphos	127 > 109 (10)	127 > 95 (15)	192 > 127 (10)
158.	Mirex	273>238 (15)	273>236 (15)	271>236 (15)
159.	Molinate	126>55 (10)	126>83 (5)	126>98 (5)
160.	Myclobutanil	179 > 125 (10)	179 > 90(30)	150 > 123 (15)
161.	Napropamide	128 > 72 (5)	128 > 100 (10)	271 > 72 (15)
162.	Nitrofen	202 > 139 (20)	283 > 253 (10)	283 > 202 (10)
163.	o.p' DDT	235 > 165 (20)	237 > 165 (20)	235 > 199 (15)
164.	Oxyfluorfen	252 > 196 (20)	252 > 146 (30)	300 > 223 (15)
165.	p,p' DDT	235 > 165 (20)	237 > 165 (20)	235 > 199 (15)
166.	p.p' DDD	235 > 165 (20)	237 > 165 (20)	235 > 199 (15)
167.	p.p' DDE	246 > 176 (30)	316 > 246 (15)	318 > 246 (15)
168.	Paclobutrazol	236 > 125 (10)	125 > 89 (20)	236 > 167 (10)
169.	Paraoxon	109 > 81 (10)	109 > 91 (5)	149 > 119 (5)
170.	Paraoxon methyl	109 > 79 (5)	230 > 106 (15)	230 > 136 (5)
171.	Parathion	139 > 109 (5)	291 > 109 (10)	139 > 81 (15)
172.	Parathion − methyl	269 > 109 (10)	125 > 47 (10)	125 > 79 (5)
173.	Pebulate	12>72 (5)	128>57 (10)	161>128 (5)

No.	Active substance	MRM transitions m/z (collision energy eV)		
		**Quantitative ion pairs**	**Qualitative ion pairs I**	**Qualitative ion pairs II**
174.	Penconazole	248 > 192 (15)	248 > 157 (25)	159 > 89 (35)
175.	Pendimethalin	252>162 (25)	252 > 161 (15)	162 > 161 (10)
176.	Pentachloroaniline	262>192 (20)	264>194 (20)	266>194 (20)
177.	Permethrin	183 > 168 (10)	183 > 165 (10)	183 > 153 (15)
178.	Phenothrin	183>168 (10)	183>155 (5)	122>81 (5)
179.	Phenthoate	274 > 121 (10)	274 > 125 (15)	121 > 77 (25)
180.	Phorate	121 > 65 (10)	121 > 47 (30)	129 > 65 (15)
181.	Phosalone	182 > 111 (15)	182 > 102 (15)	182 > 75 (30)
182.	Phosmet	160 > 77 (20)	160 > 133 (10)	160 > 105 (15)
183.	Phthalimide	147>103 (5)	147>76 (25)	104>76 (10)
184.	Picoxystrobin	145 > 102 (25)	145 > 115 (15)	145 > 117 (10)
185.	Pirimicarb	238 > 166 (10)	166 > 55 (20)	166 > 96 (15)
186.	Pirimiphos − methyl	290 > 125 (20)	233 > 151 (5)	233 > 125 (5)
187.	Pirimiphos ethyl	318 > 166 (10)	318 > 182 (10)	152 > 84 (10)
188.	Prochloraz	196 > 97 (30)	180 > 138 (10)	310 > 70 (15)
189.	Procymidone	96 > 67 (10)	96 > 53 (15)	283 > 96 (10)
190.	Profenofos	208 > 63 (30)	339 >269 (15)	299 > 269 (5)
191.	Propachlor	120 > 77 (10)	176 > 57 (10)	120 > 92 (10)
192.	Propaquizafop	163 > 100 (25)	163 > 136 (10)	299 > 91 (20)
193.	Propargite	135 > 107 (10)	150 > 135 (5)	135 > 77 (30)
194.	Propazine	214 > 172 (10)	229 > 58 (10)	229 > 214 (5)
195.	Propham	119 > 91 (10)	137 > 93 (10)	179 > 93 (15)
196.	Propiconazole	173 > 145 (15)	173 > 109 (30)	173 > 74 (45)
197.	Propoxur	110 > 63 (25)	110 > 64 (15)	152 > 110 (10)
198.	Propyzamide	173 > 145 (15)	175 > 147 (15)	173 > 109 (30)
199.	Prosulfocarb	128 > 86 (0)	251 > 86 (10)	251 > 128 (10)
200.	Prothiofos	113 > 95 (10)	267 > 239 (5)	309 > 239 (15)
201.	Pyraclostrobin	132 >77.1 (20)	164 > 132 (10)	132 > 104 (15)
202.	Pyrazophos	221 > 193 (10)	232 > 204 (10)	221 > 149 (15)
203.	Pyridaben	147 > 117 (20)	147 > 132 (10)	147 > 105 (10)
204.	Pyrimethanil	198 > 183 (15)	198 > 118 (35)	198 > 158 (20)
205.	Pyriproxyfen	136 > 78 (20)	136 > 96 (15)	321 > 222 (10)
206.	Quinalphos	146 > 118 (10)	146 > 91 (30)	157 > 129 (15)
207.	Quinoxyfen	237 > 208 (30)	272 > 237 (10)	307 > 237 (20)
208.	Quintozene	295>265 (10)	295>237 (15)	265>237 (10)

No.	Active substance	MRM transitions m/z (collision energy eV)		
		**Quantitative ion pairs**	**Qualitative ion pairs I**	**Qualitative ion pairs II**
209.	Quizalofop−P−ethyl	372 > 299 (10)	163 > 136 (10)	163 > 100 (20)
210.	Resmethrin	123>81 (5)	171>128 (5)	171>143 (15)
211.	Simazine	201 > 173 (5)	173 > 172 (5)	173 > 138 (5)
212.	Spirodiclofen	109 > 81 (10)	109 > 79 (15)	312 > 259 (10)
213.	Spiromesifen	272 > 524 (5)	272 > 209 (10)	273 > 255 (5)
214.	Spiroxamine	100 > 58 (10)	100 > 72 (5)	198 > 126 (5)
215.	Sulfotep	201>145 (10)	321>201 (10)	321>145 (25)
216.	Tebuconazole	125 > 89 (15)	250 > 125 (20)	125 > 99 (20)
217.	Tebufenpyrad	276 > 171 (10)	333 > 171 (15)	318 > 131 (15)
218.	Tecnazene	261 > 203 (20)	215 > 179 (5)	259 > 201 (5)
219.	Tefluthrin	177 > 127 (15)	197 > 141 (10)	177 > 87 (30)
220.	Terbacil	160>117.1 (5)	161>144 (10)	160>76 (15)
221.	Terbufos	230>175 (10)	230>129 (20)	152>97 (5)
222.	Terbufos sulfone	152> 96(10)	124>96 (5)	198>143 (10)
223.	Terbuthylazine	173 > 172 (5)	229 > 173 (5)	173 > 138 (5)
224.	Tetrachlorvinfos	329 > 109 (15)	331 > 109 (15)	109 > 79 (5)
225.	Tetraconazole	171 > 136 (10)	336 > 218 (20)	336 > 204 (30)
226.	Tetradifon	159 > 131 (10)	227 > 199 (15)	159 > 111 (20)
227.	Tetrahydrophthalimide (THPI)	79.0>77.0 (15)	79.0>51.0 (25)	151.0>80.0 (5)
228.	Tetramethrin	164 >107 (10)	164 > 77 (25)	123 > 81 (10)
229.	Thionazin	143>79 (10)	175>79 (10)	107>79 (15)
230.	Tolclofos − methyl	265 > 250 (15)	265 > 93 (25)	125 > 47 (15)
231.	Tolfenpyrad	211>210 (10)	197>154 (10)	383>171 (25)
232.	Tolylfluanid	137 > 91 (20)	238 > 137 (15)	137 > 65 (30)
233.	Tralomethrin	252>93 (15)	181>152 (25)	250>172 (5)
234.	Triadimefon	208 > 181 (5)	208 > 111 (20)	128 > 65 (20)
235.	Triadimenol	128 > 65 (25)	168 > 70 (10)	128 > 100 (10)
236.	Triallate	268>184 (20)	270>186 (20)	268>226 (10)
237.	Triazophos	161 > 134 (5)	161 > 106 (10)	161 > 91 (15)
238.	Trifloxystrobin	116 > 89 (15)	116 > 63 (30)	131 > 89 (15)
239.	Triflumizole	206 > 179 (15)	206 > 186 (10)	179 > 144 (15)
240.	Trifluralin	306 > 264 (5)	264 > 160 (15)	264 > 206 (5)
241.	Triticonazole	235 > 217 (5)	235 > 182 (10)	217 > 165 (25)

No.	Active substance	MRM transitions m/z (collision energy eV)		
		**Quantitative ion pairs**	**Qualitative ion pairs I**	**Qualitative ion pairs II**
242.	Vinclozolin	125 > 47 (15)	125 > 79 (5)	286 > 93 (20)
243.	Zoxamide	187 > 159 (15)	189 > 161 (15)	187 > 123 (30)
244.	α−endosulfan	195 > 159 (5)	195 > 160 (5)	195 > 125 (20)
245.	α−HCH	217 > 181 (5)	219 > 183 (5)	181 > 145 (15)
246.	β−endosulfan	207 > 172 (15)	195 > 159 (10)	195 > 125 (25)
247.	β−HCH	181 > 145 (15)	217 > 181 (5)	219 > 183 (5)
248.	γ− cyhalothrin	197 > 141 (10)	181 > 152 (25)	197 > 161 (5)
249.	γ−HCH (lindane)	217 > 181 (5)	181 > 145 (15)	219 > 183(5)
250.	δ−HCH	217>181 (5)	181>145 (15)	219 >183 (5)
251.	λ−cyhalothrin	197 > 141 (10)	181 > 152 (25)	197 > 161 (5)
252.	τ−fluvalinate	250 > 55 (40)	181 > 152 (40)	250 > 200 (40)
253.	Triphenyl phosphate (TPP) (IS)	215 > 168 (15)	232 > 215 (10)	326 > 325 (5)

Table S5. Short−term risk assessment equations for particular vegetable commodities.

Case	Vegetable commodity	Equation for calculation of international short−term intake (IESTI)
1	Basil and edible flowers<br>Celery leaves<br>Garlic<br>Parsley	IESTI= [LP×HR×PF×CF]/bw
2a	Tomato	IESTI=[U×HR×PF×CF+(LP−U)×HR×PF×CF]/bw
2b	Broccoli<br>Chinese cabbage<br>Cucumbers<br>Leeks<br>Lettuces<br>Onions<br>Potatoes<br>Radishes<br>Sweet potatoes<br>Sweet peppers	IESTI= [LP×HR×PF×CF×VF]/bw

IESTI − International Estimated Short−Term Intake ;
LP − Large Portion (97.5th percentile of eaters) (g kg^{-1} bw);
HR − Highest Residue according to residue definition for enforcement in composite sample (mg kg^{-1});
CF − Conversion Factor residue definition enforcement to residue definition risk assessment;
PF − Processing Factor (calculated as the ratio of residues in processed product, divided by residue concentration in unprocessed product);
VF − Variability Factor VF=1 when U< 25 g; VF = 7 when U between 25 and 250 g; VF = 5 when U above 250g;
bw − body weight for the subgroup of the population related to the LP or mean consumption (kg).

Table S6. Consumption data of particular vegetable commodities for the most critical sub–population of children and adults.

Vegetable commodity	Children			Adults		
	MS critical diet	Body weight (kg)	Large portion (g/person)	MS critical diet	Body weight (kg)	Large portion (g/person)
Basil and edible flowers	DE children	16.15	11.8	NL general population	65.8	8.1
Broccoli	BE toddlers	17.8	148.1	NL general population	65.8	424.5
Celery stalk	NL toddler	10.2	4.9	NL general population	65.8	21.4
Chinese cabbage	BE toddlers	17.8	114.4	UK 15-18 years old	63.8	323.0
Cucumber	CZ 4–6 years old	21.4	280.5	NL general population	65.8	365.8
Garlic	IE children	20.0	70.6	UK vegetarians	66.7	43.0
Lettuce	NL children	18.4	140.1	NL general population	65.8	159.8
Leek	BE toddlers	17.8	149.9	IE adults	75.2	145.7
Onion	BE toddlers	17.8	57.8	UK vegetarians	66.7	141.5
Parsley	UK 7–10 years old	30.9	33.8	UK vegetarians	66.7	79.9
Potato	UK infants	8.7	191.1	UK vegetarians	66.7	694.4
Radish	NL children	18.4	64.4	DE women 14–50 years old	67.5	100.5
Sweet pepper	DE children	16.15	137.3	UK vegetarians	66.7	155.4
Sweet potato	IE children	20.0	15.1	IE adults	75.2	266.0
Tomato	BE toddlers	17.8	180.0	LT adults	70	450.0
BE – Belgium, CZ– Czech, DE – German, IE – Irish, LT – Lithuanian, NL – Dutch, UK – British						

Table S7. Detailed summary of detected pesticides in selected vegetable samples.

No	Assortment	Country of origin	Pesticide	Concentration (mg/kg)	Pesticide	Concentration (mg/kg)	Pesticide	Concentration (mg/kg)	Pesticide	Concentration (mg/kg)	Pesticide	Concentration (mg/kg)
Cabbage vegetables												
105	broccoli	Spain	Fluopyram (F)	0.005	Spirotetramat (I)	0.044						
31	broccoli	Spain	bdl	bdl								
334	broccoli	Italy	Azoxystrobin (F)	0.025	Difenoconazole (F)	0.012						
112	Chinese cabbage	Poland	Boscalid (F)	0.005	*Flonicamid (I)*	*0.10*						
220	radish	Italy	**Chlorpyrifos methyl (I)**	**0.008**	**Dimethomorph (F)**	**0.033**	Fludioxonil (F)	0.005	Fluopyram (F)	0.005		
294	radish	Italy	**Dimethomorph (F)**	**0.005**								
32	radish	Italy	Mandipropamid (F)	0.020								
Celery vegetable												
108	celery stalk	Spain	Azoxystrobin (F)	0.005	Boscalid (F)	0.005	Difenoconazole (F)	0.024	Fluopicolide (F)	0.005	Spirotetramat (I)	0.005
211	parsley leaves	Spain	Azoxystrobin (F)	0.22	Boscalid (F)	0.005	Difenoconazole (F)	0.25	Fludioxonil (F)	0.005	Propamocarb (F)	0.87
Fruiting vegetable												
34	cucumber	Poland	Boscalid (F)	0.005	Cyprodinil (F)	0.005	Fluopyram (F)	0.005	Pyrimethanil (F)	0.015		
54	cucumber	Türkiye	Acetamiprid (I)	0.034	**Chlorpyrifos methyl (I)**	**0.005**	Cyjazofamid (F)	0.005	Cyprodinil (F)	0.005	Flonicamid (I)	0.127
			Propamocarb (F)	0.016								
243	cucumber	Poland	Fluopyram (F)	0.067	Propamocarb (F)	0.005						
277	cucumber	Poland	Azoxystrobin (F)	0.006	Fluopicolide (F)	0.005	Propamocarb (F)	0.043				
Leaf vegetables, herbs and edible flowers												
141	lettuce	Spain	Acetamiprid (I)	0.11	Ametoctradin (F)	0.012	Fluopicolide (F)	0.005	Spirotetramat (I)	0.036		
204	basil	Poland	Azoxystrobin (F)	0.005	Boscalid (F)	0.005	Fluopicolide (F)	0.005	Propamocarb (F)	0.005	Pyrimethanil (F)	0.005
Nightshade vegetable												
136	eggplant	Spain	Abamectin* (I)	0.006	Acetamiprid (I)	0.083	Metrafenone (F)	0.005				
73	potato	Poland	Fludioxonil (F)	0.005								
129	potato	Cyprus	Difenoconazole (F)	0.005	Fludioxonil (F)	0.005	Fluopicolide (F)	0.005	**Flutriafol (F)**	**0.005**	Propamocarb (F)	0.005
368	potato	Poland	Propamocarb (F)	0.005								
37	potato	Poland	bdl	bdl								

382	potato	Poland	Fluxapyroxad (F)	0.010	**Imidacloprid (I)**	**0.006**	Propamocarb (F)	0.007				
85	red pepper	Türkiye	Boscalid (F)	0.005	Flonicamid (I)	0.11	Fludioxonil (F)	0.005	Pirydalil (I)	0.092		
94	sweet potato	USA	Boscalid (F)	0.005	Fludioxonil (F)	1.1						
25	sweet potato	Spain	bdl	bdl								
394	sweet potato	USA	**Dicloran (F)**	**0.010**	Fludioxonil (F)	0.330						
46	tomato	Spain	Propamokarb (F)	**0.005**	Cyazofamid (F)	0.005						
62	tomato	Italy	**Benthiavalicarb isopropyl (F)**	**0.095**	Boscalid (F)	0.005	Difenoconazole (F)	0.031	Emamectin* (I)	0.005	Fluopyram (F)	0.027
			Penconazole (F)	0.020	Tetraconazole (F)	0.005						
22	tomato	Poland	Boscalid (F)	0.23	Cyprodinil (F)	0.005	Fludioxonil (F)	0.005	Fluopyram (F)	0.065	Pyraclostrobin (F)	0.014
			Pyrimethanil (F)	0.013								
237	tomato	Poland	Azoxystrobin (F)	0.082	Captan (F)	0.13	Deltamethrin (I)	0.005	Flonicamid (I)	0.037	Penthiopyrad (F)	0.28
			Propamocarb (F)	0.41	Pyrimethanil (F)	0.007	Spirotetramat (I)	0.005	Tebuconazole (F)	0.082		
261	tomato	Morocco	Cyprodinil (F)	0.021	Flonicamid (I)	0.020	Fludioxonil (F)	0.022	Fluopyram (F)	0.018	Mandipropamid (F)	0.005
			Tebuconazole (F)	0.021	Trifloxystrobin (F)	0.031						
302	tomato	Germany	**Diafenthiuron (I)**	**0.005**	**Fenbutatin oxide (I)**	**0.005**	Sulfoxaflor (I)	0.009				
34	tomato	Netherlands	Fluopyram (F)	0.008	Pyridalyl (I)	0.005	Spinosad* (I)	0.036				
						Onion vegetable						
193	garlic	Spain	Azoxystrobin (F)	0.005	Boscalid (F)	0.36	**Prochloraz (F)**	**0.014**	Pyraclostrobin (F)	0.086	Tebuconazole (F)	0.005
152	leek	Belgium	Ametoctradin (F)	0.14	Boscalid (F)	0.005	**Dimethomorph (F)**	**0.029**	Fludioxonil (F)	0.005	Fluopicolide (F)	0.007
			Pyrimethanil (F)	0.005	Propamocarb (F)	0.007	Prothioconazole (F)	0.005	Tebuconazole (F)	0.005		
161	onion	India	bdl	bdl								
170	onion	Poland	Boscalid (F)	0.005	Spirotetramat (I)	0.006						
184	onion	Netherlands	bdl	bdl								
286	onion	France	Azoxystrobin (F)	0.012	Boscalid (F)	0.048	Cyjazolamid (F)	0.005	Fluopicolide (F)	0.013	Propamocarb (F)	0.009
			Pyraclostrobin (F)	0.014								
357	onion	Spain	bdl	bdl								

F: Fungicide; I: Insecticide; bdl: below detection limit; the bold name of the compound indicates that it is not approved in the EU**;** the name of the compound in italics indicates that the MRL value is exceeded;

*Abamectin (sum of avermectin B1a, avermectin B1b and delta−8,9 isomer of avermectin B1a, expressed as avermectin B1a), *emamectin (sum of emamectin B1a and emamectin B1b expressed as emamectin), spinosad (spinosad, sum of spinosyn A and spinosyn D).
